# Butyrate ameliorates doxorubicin-induced heart failure by inhibiting cardiomyocyte ferroptosis through the gut-heart axis

**DOI:** 10.1016/j.isci.2026.114754

**Published:** 2026-01-20

**Authors:** Shibin Zeng, Qing Xie, Rong Zhang, Ting Yang, Dan Liu, Jiani Zhang, Shudong Ma, Xiaozhong Qiu

**Affiliations:** 1 Department of Oncology, Nanfang Hospital, Southern Medical University, Guangzhou, Guangdong 510515, P.R. China; 2 International Center for Translational Medicine, The Eighth Affiliated Hospital, Southern Medical University (The First People’s Hospital of Shunde), Foshan, Guangdong 528300, P.R. China; 3 Guangdong Provincial Key Laboratory of Construction and Detection in Tissue Engineering, School of Basic Medical Science, Southern Medical University, Guangzhou, Guangdong 510515, P.R. China; 4 Department of Oncology, The Fifth Affiliated Hospital of Southern Medical University, Guangzhou, Guangdong 510900, P.R. China; 5 Department of Oncology, Guangzhou Fuda Cancer Hospital, Guangzhou, Guangdong 510665, P.R. China

**Keywords:** Pharmacology, Microbiome, Metabolomics

## Abstract

Doxorubicin (DOX), a widely used chemotherapeutic agent, is clinically limited by DOX-induced heart failure (DIHF). Emerging evidence links gut microbial dysbiosis to exacerbating DIHF progression, yet the mechanisms remain elusive. Herein, we established a rat DIHF susceptibility model to investigate the gut microbiota’s regulatory role. Multi-omics analyses indicated that DIHF severity was associated with reduced butyrate-producing bacteria and systemic butyrate levels. Sodium butyrate (NaB) significantly alleviated DOX-induced cardiomyocyte toxicity and DIHF. Mechanistically, NaB strengthened the colonic and cardiac barrier functions and reduced gut microbiota translocation to the heart and cardiac lipopolysaccharide (LPS) accumulation. NaB altered cardiac bacterial composition and functions, reduced cardiac Fe^2+^ levels, and inhibited cardiomyocyte ferroptosis. Further results confirmed that NaB mitigated DOX-induced ferroptosis via the GPX4/GSH pathway. Collectively, this study indicated that butyrate ameliorates DIHF by inhibiting cardiomyocyte ferroptosis through the gut-heart axis, providing translational potential for microbiota-targeted cardioprotective strategies in DIHF.

## Highlights

• Butyrate-producing bacteria and butyrate play a critical role in DIHF susceptibility
• We first identified resident bacteria in DOX-treated cardiac tissue
• Butyrate lowers cardiac translocation of gut microbiota, alters cardiac bacterial composition
• Butyrate inhibits DOX-induced cardiomyocyte ferroptosis via the GPX4/GSH pathway

## Graphical abstract

**Figure:**
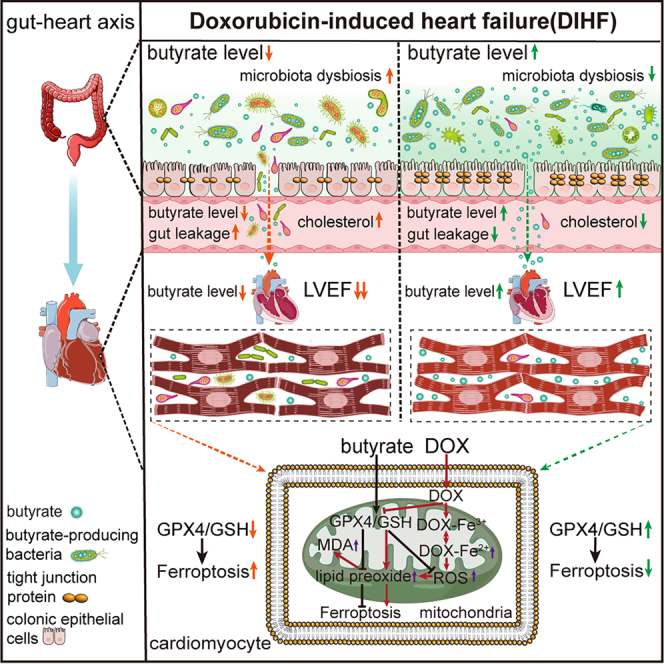

## Introduction

Heart failure (HF) represents a significant global health burden, characterized by high morbidity and mortality rates.^1^^,^^2^^,^^3^ The etiology of HF is multifactorial; many anticancer therapies pose a high risk of cardiotoxicity, exacerbating heart dysfunction and presenting a significant challenge in drug development and clinical practice.^4^^,^^5^^,^^6^^,^^7^ HF induced by anticancer therapies manifests as a severe cardiovascular complication, drawing significant attention in the realm of cardio-oncology.^8^ Effective intervention of Cancer therapy-related cardiovascular toxicity (CTR-CVT) remarkably improves the prognosis of patients with cancer with HF,^8^ however, one of the major challenges of the CTR-CVT stems from the complexity of its mechanisms that drive CTR-CVT progression to HF.^9^^,^^10^^,^^11^ Therefore, gaining a comprehensive understanding of the fundamental mechanisms of CTR-CVT holds great significance.

Doxorubicin (DOX), as one of the most commonly used chemotherapeutic agents for multiple cancers, can cause dose-dependent CTR-CVT.^12^ Consequently, DOX-induced heart failure (DIHF) represents a prevalent complication within clinical oncology. Recent researches have indicated that DOX-induced cardiotoxicity (DIC) may be linked to ferroptosis, apoptosis, autophagy, and pyroptosis in cardiomyocytes;^13^^,^^14^^,^^15^^,^^16^ however, the underlying mechanisms driving the progression of DIHF remain inadequately explored. Notably, growing evidence suggests chemotherapeutic agents may amplify their toxicity through inducing gut microbiota dysbiosis (GMD).^17^^,^^18^^,^^19^ The gut-heart axis, composed of gut microbiota and their metabolites, the gut barrier, and cardiac tissues, plays a crucial regulatory role in cardiovascular diseases (CVDs).^20^^,^^21^ Furthermore, researchers have established an association between GMD, its metabolic profiles, and the progression of HF.^22^^,^^23^^,^^24^ However, the mechanisms of chemotherapy-induced GMD exacerbating cardiotoxicity remain poorly understood. Therefore, an in-depth exploration of the clinically prevalent chemotherapeutic agent DOX and its effects on the progression of DIHF according to the gut-heart axis is essential for improving clinical outcomes of patients with cancer.

In this study, we first demonstrated a significant correlation between the severity of DIHF and the differences in gut microbiota composition and butyrate level. Specifically, within the same DOX dosage model, there were two distinct phenotypes: DIHF-High Susceptibility (DIHF-HS) rats that developed severe DIHF, and DIHF-Low Susceptibility (DIHF-LS) rats that exhibited milder DIHF. The phenotypes of susceptibility to DIHF were further validated through fecal microbiota transplantation (FMT) from donor rats with DIHF-LS and -HS to recipient rats. Multi-omics analyses indicated that butyrate-producing bacteria and butyrate play a crucial role in reducing the susceptibility to DIHF. Furthermore, NaB effectively reduced cardiomyocyte toxicity *in vitro* and ameliorated DIHF *in vivo*. Mechanistically, NaB supplementation regulated gut microbiota composition and function, alleviating GMD via multi-omics analyses. Notably, we first identified the presence of resident bacteria in DOX-treated cardiac tissue, and NaB significantly reduced the bacteria's abundance. For this reason, NaB enhanced barrier functions of colonic and cardiac tissues, reducing gut bacterial translocation and LPS accumulation in the heart. NaB altered cardiac bacterial composition and functions, leading to Fe^2+^ level decrease in favor of inhibiting ferroptosis. We further confirmed that NaB significantly suppressed DOX-induced cardiomyocyte ferroptosis through the GPX4/GSH pathway *in vitro* and *in vivo*. In summary, this study indicated that butyrate ameliorated the DIHF by inhibiting cardiomyocyte ferroptosis via the microbiota/metabolites-gut-heart axis, highlighting its potential as a cardioprotective strategy against the DIHF.

## Results

### The gut microbiota plays a crucial role in modulating the doxorubicin-induced heart failure

**Figure 1:**
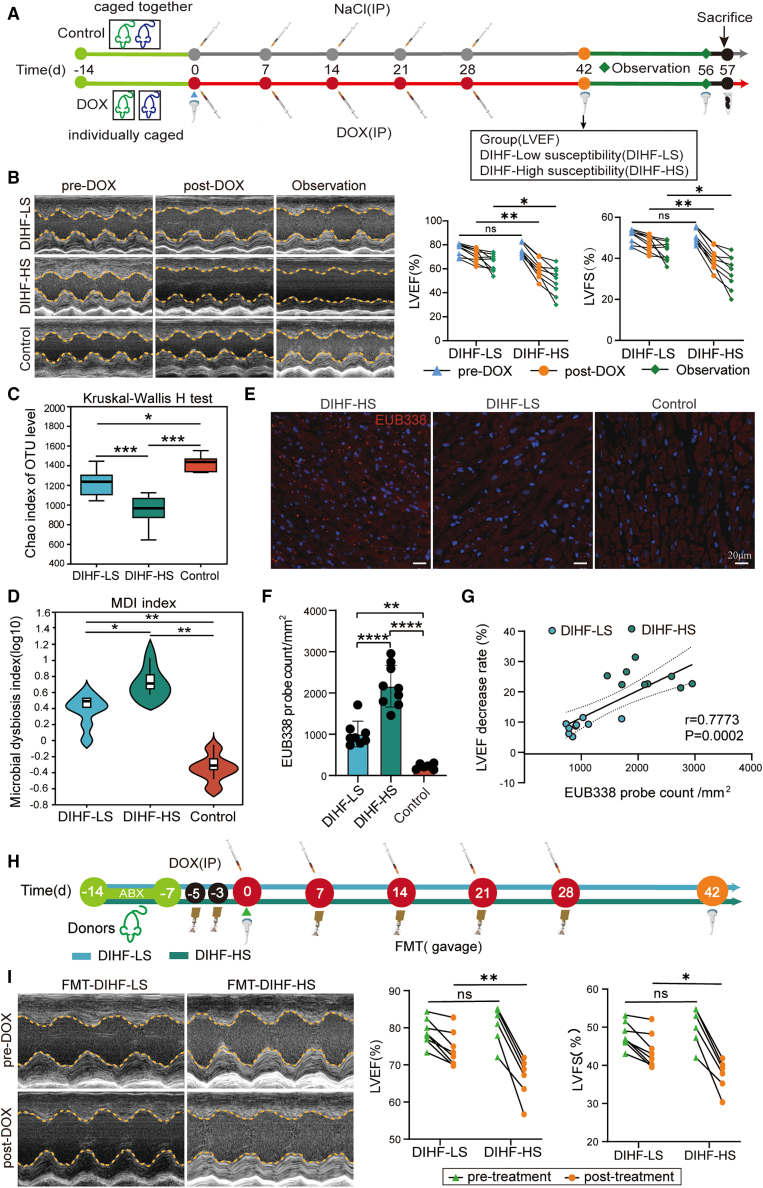
The gut microbiota plays a crucial role in modulating the DIHF (A) The schematic diagram shows the experimental design and timeline of the rat DIHF model. (B) Representative echocardiograms and quantitative analysis of LVEF (%), LVFS (%) in the DIHF-LS (*n* = 8), DIHF-HS (*n* = 9), Control (*n* = 6) groups. (C) ɑ-diversity (Chao index) of gut microbiota derived from the DIHF-LS, DIHF-HS, and control groups by 16S rRNA V3-V4 NGS. (D) Microbiota dysbiosis index (MDI) of gut microbiota derived from the DIHF-LS, DIHF-HS, and control groups. (E and F) Representative images (E), quantitative analysis (F) of EUB338 bacterial probe count in cardiac tissue of the DIHF-LS, DIHF-HS, and control groups via FISH (scale bars, 20 μm). (G) The Pearson’s correlation analysis of EUB338 probe count and LVEF decrease rate in the DIHF-LS and DIHF-HS groups. Statistical significance was determined using the simple linear regression of Correlation. (H) The schematic illustration for the experimental design and timeline of FMT from the DIHF-LS and DIHF-HS groups. (I) Representative echocardiograms and quantitative analysis of LVEF (%), LVFS (%) of FMT-DIHF-LS, FMT-DIHF-HS group (*n* = 8 per group) by echocardiographic analysis. Error bars represent the mean ± SEM. For normally distributed data with homogeneous variances, Statistical analyses were performed using Student’s *t* tests for two groups and one/two-way ANOVA for multiple comparisons. With nonnormal distribution or unequal variances, the Wilcoxon rank-sum test was applied for two groups and the Kruskal-Wallis test for three groups. ∗*p* < 0.05; ∗∗*p* < 0.01; ∗∗∗*p* < 0.001; and ∗∗∗∗*p* < 0.0001.

To explore the correlation of gut microbiota with DIHF, we caged rats separately to induce individual differences in gut microbiota, followed by establishing the DIHF model. Echocardiography was performed to measure the baseline cardiac function, LVFS, and LVEF before DOX treatment and then repeated to assess the changes in cardiac function after DOX treatment. These results revealed that rats exhibited significant differences in the severity of DIHF within the same DOX dosage. DIHF-High Susceptibility (DIHF-HS, LVEF decrease >30%) rats developed severe heart failure, and DIHF-Low Susceptibility (DIHF-LS, LVEF decrease <20%) rats exhibited milder heart failure (Figure 1A). Furthermore, echocardiography was again performed to assess cardiac function after a 2-week observation period without DOX treatment. The finding indicated that the degree of decrease in LVEF was considerably greater in the DIHF-HS group than in the DIHF-LS group (Figure 1B). This result indicated that the DIHF susceptibility may be associated with differences in gut microbiota composition. To probe the reason, the rat feces were subjected to 16S rRNA V3-V4 sequencing, and the results demonstrated that the ɑ diversity of the DIHF-HS rats was markedly reduced compared to that of the DIHF-LS and the control group (Figure 1C). Principal component analysis (PCA) indicated that the gut microbiotic composition remarkably differed between groups on the genus level (Figure S1A) and family level (Figure S1B). The microbiota dysbiosis index (MDI) was markedly increased in the DIHF-HS group compared to the DIHF-LS and control groups (Figure 1D). These findings implied that the MDI is positively correlated with susceptibility to DIHF. The Linear discriminant analysis effect size (LEfSe) analysis was performed to further identify the different microbiota among the three groups. On the family level (Figure S1C), the abundance of *Lachnospiraceae, Bacteroidaceae,* and *Saccharimonadaceae* was remarkably elevated in the DIHF-LS group in comparison with the other groups, implying that these bacteria provide potential cardioprotection against DIHF. The abundance of *Lactobacillaceae, Helicobacteraceae,* and *Atopobiaceae* was remarkably elevated in the DIHF-HS group compared to the other groups, implying that they are potentially associated with the exacerbation of DIHF. Similarly, on the genus level (Figure S1D), the abundance of *Blautia, unclassified_f_Lachnospiraceae, Ruminococcus, Marvinbryantia, Bacteroides, Candidatus_Saccharimonas, and Lachnoclostridium* were remarkably elevated in the DIHF-LS group in contrast to the other groups, implying that they provided potential cardioprotection against DIHF. The abundance of *Lactobacillus, Helicobacter, Coriobacteriaceae_UCG-002, and Roseburia* was remarkably elevated in the DIHF-HS group in contrast to the other groups, implying that they are potentially associated with the exacerbation of DIHF.

In the context of HF, gut bacteria translocate into the bloodstream through gut leakage. We explored whether gut bacteria translocated to cardiac tissues and whether this translocation was associated with susceptibility to DIHF. The universal bacterial probe EUB338(sequence: 5′-GCTGCCTCCCGTAGGAGT-3′) was used to detect cardiac resident bacteria via FISH, and the result first demonstrated the presence of resident bacteria in DIHF cardiac tissues (Figure 1E), confirming the occurrence of bacteria translocation to the heart. Importantly, quantitative analysis revealed a notably amplified EUB338 probe count in the DIHF-HS group than in the DIHF-LS group (Figure 1F). To demonstrate the correlation between cardiac resident bacteria and susceptibility to DIHF, Pearson’s correlation analysis was conducted, and the result revealed a notable positive correlation between cardiac resident bacteria and susceptibility to DIHF (Figure 1G), suggesting that cardiac resident bacteria might contribute to the development of DIHF.

To further validate the relation of gut microbiota with susceptibility to DIHF, FMT from a donor rat in the DIHF-LS and DIHF-HS groups to recipient rats was performed (Figure 1H), and the results revealed that LVFS and LVEF were significantly decreased in the FMT-DIHF-HS group than in the FMT-DIHF-LS group (Figure 1I). These findings indicate that the gut microbiota plays a crucial role in modulating DIHF.

### Butyrate-producing bacteria and butyrate levels are significantly reduced, and lipid metabolism is dysregulated in the doxorubicin-induced heart failure-HS rats

**Figure 2:**
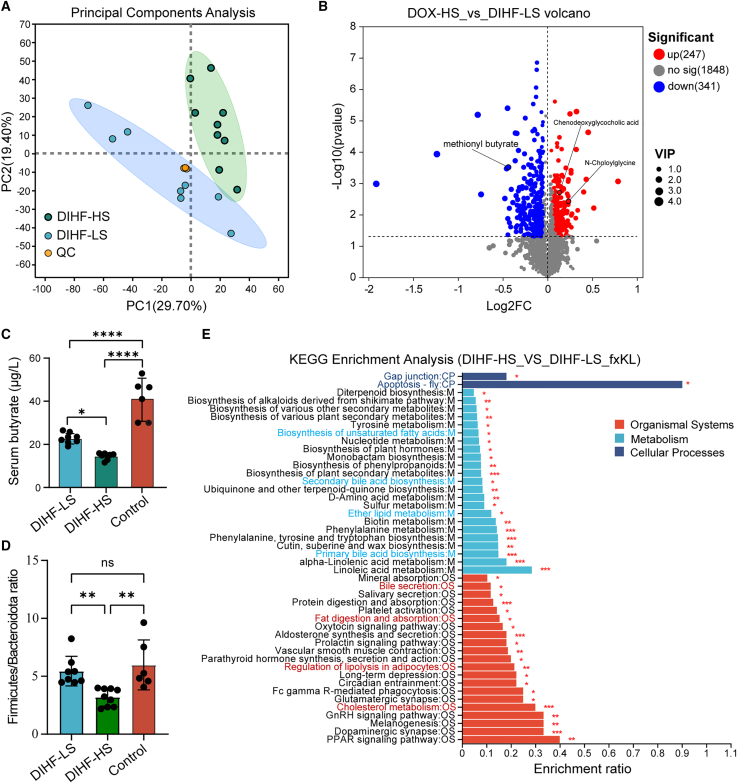
The abundance of butyrate-producing bacteria and butyrate levels were significantly reduced, and lipid metabolism was dysregulated in the DIHF-HS rats (A) Principal component analysis (PCA) of fecal non-targeted metabolomics (LC-MS) profiling in the DIHF-HS and the DIHF-LS group. (B) A volcano plot shows differential abundance of fecal metabolites in the DIHF-HS and the DIHF-LS group (significant *p* < 0.05 and OPLS-DA VIP >1). (C) Serum butyrate concentration (μg/mL) was determined by colorimetric assay in the DIHF-LS, DIHF-HS, and control group. (D) *Firmicutes/Bacteroidota* ratio of the DIHF-LS, DIHF-HS, and control group. (E) KEGG enrichment analysis of the DIHF-HS group, compared with the DIHF-LS group. DIHF-HS group *n* = 9, DIHF-LS group *n* = 8, Control group *n* = 6. Error bars represent the mean ± SEM. For normally distributed data with homogeneous variances, statistical analyses were performed using one/two-way ANOVA for multiple comparisons. For non-normal/heteroscedastic data, the Wilcoxon rank-sum test was applied for two groups. ∗*p* < 0.05; ∗∗*p* < 0.01; ∗∗∗*p* < 0.001; and ∗∗∗∗*p* < 0.0001.

Since the gut microbiota plays a pivotal role in the susceptibility to DIHF, we next investigated whether altered gut microbiota was accompanied by alterations in its metabolites and functions. Feces from the DIHF-HS group and DIHF-LS group were subjected to an untargeted metabolomics LC-MS assay. PCA revealed that the metabolite compositions were remarkably different between the two groups (Figure 2A). A volcano plot revealed that the abundances of 247 gut metabolites significantly increased, whereas those of 341 decreased. Among the metabolites, the abundance of the butyrate derivative methionyl butyrate was remarkably decreased in the DIHF-HS group (Figure 2B). In addition, the abundances of PAGln (Figure S2A) and TMAO (Figure S2B), which are two major gut metabolites that affect cardiac function, were not considerably different between the two groups. We further measure the butyrate concentration in each rat of the three groups via a colorimetric method. Consistently, we found that the butyrate levels in the DIHF-HS group rats were markedly lower than those in the DIHF-LS and control groups (Figure 2C). Moreover, the Firmicutes/Bacteroidetes ratio, an index for evaluating the potential of butyrate-producing gut microbiota, was remarkably lower in the DIHF-HS group rats than in the DIHF-LS group and the control group rats (Figure 2D). Notably, as shown in Figure 1G, in the DIHF-LS group, the abundance of *Blautia, unclassified_f_Lachnospiraceae, Ruminococcus, and Lachnoclostridium*, all of which belong to *Lachnospiraceae*, was remarkably elevated, and these were the main butyrate-producing gut microbiota. Whereas, only *Roseburia* was found to be a butyrate-producing bacterium, which further accounted for the decrease of butyrate levels in the DIHF-HS group rats.

Regarding the alterations in metabolite functions, the gut metabolites were associated with lipid metabolism and cardiovascular diseases in the DIHF-HS group via KEGG pathway analysis (Figure S2C). Moreover, the metabolite enrichment rates of pathways including gap junctions, apoptosis, unsaturated fatty acids, biosynthesis of primary and secondary bile acids, fat digestion and absorption, cholesterol metabolism, and bile secretion were remarkably enriched in the DIHF-HS group compared with those in the DIHF-LS group via KEGG enrichment analysis (Figure 2E). The abundance of chenodeoxyglycocholic acid and N-choloylglycine was markedly higher in the DIHF-HS group, whereas taurocholic acid was notably lower (Figure 2B; Figure S2D). Taken together, reduced butyrate-producing bacteria and butyrate levels were related to high susceptibility to DIHF, accompanied by dysregulated lipid metabolism.

### Sodium butyrate protects cardiomyocytes and ameliorates doxorubicin-induced heart failure *in vitro* and *in vivo*

**Figure 3:**
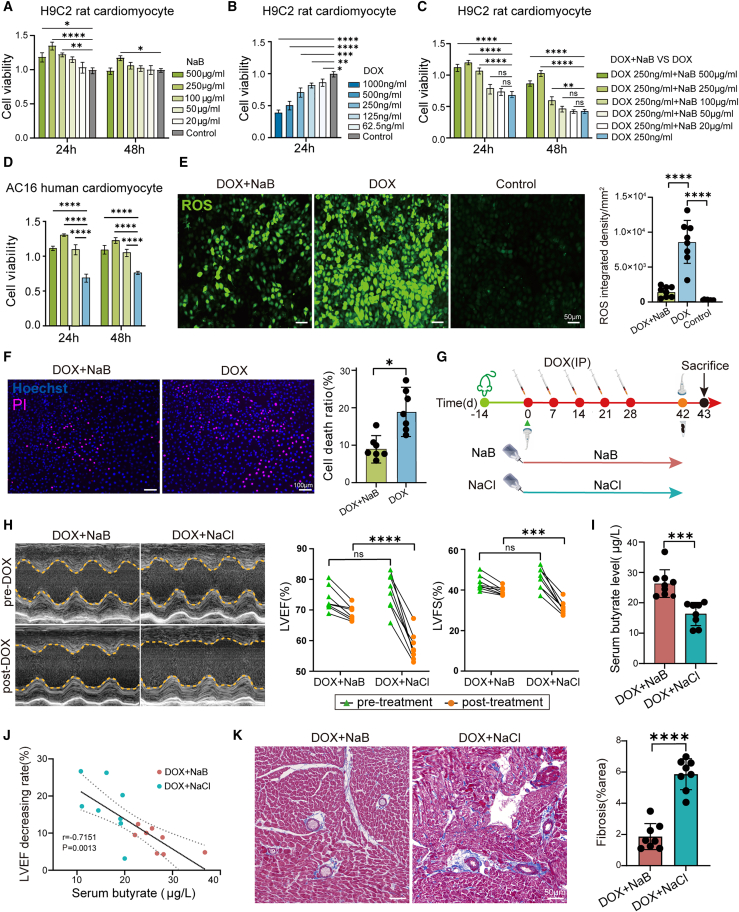
NaB reduced cardiomyocyte toxicity *in vitro* and ameliorated DIHF *in vivo* (A) Cell viability of H9C2 cardiomyocytes co-cultured with NaB by CCK-8 detection *in vitro* (*n* = 8 per group). (B) Cell viability of H9C2 cardiomyocytes co-cultured with DOX by CCK-8 detection *in vitro* (*n* = 8 per group). (C) Cell viability of H9C2 cardiomyocytes co-cultured with DOX for 24 h, followed by NaB for 24 h, 48 h by CCK-8 detection *in vitro* (*n* = 8 per group). (D) Cell viability of AC16 human cardiomyocytes co-cultured with DOX for 24 h, followed with NaB for 24 h, 48 h by CCK-8 detection *in vitro*. (E) Representative images and quantitative analysis of reactive oxygen species (ROS) in H9C2 cardiomyocytes in the DOX+NaB, DOX, and control group by DCFH-DA fluorescent probe test (*n* = 8 per group) (scale bars, 50 μm). (F) Representative images and quantitative analysis of cell death of H9C2 cardiomyocytes in the DOX+NaB, DOX group by Propidium iodide (PI) staining (*n* = 8 per group) (scale bars, 100 μm). (G) The schematic illustration for the experimental design and timeline of the DOX+NaB and DOX+NaCl groups in the DIHF model. (H) Representative echocardiograms and quantitative analysis of LVEF (%), LVFS (%) of pre-and post-DOX in the DOX+NaB and DOX+NaCl groups in the DIHF model. (I) Serum butyrate concentration (μg/mL) in the DOX+NaB and DOX+NaCl groups. (J) The Pearson’s correlation analysis of serum butyrate concentration (μg/L) and LVEF decrease rate (%) in the DOX+NaB and DOX+NaCl group. Statistical significance was determined using the simple linear regression of correlation. (K) Representative images and quantitative analysis of cardiac tissue fibrosis by Masson trichrome staining in the DOX+NaB and DOX+NaCl groups (scale bars, 50 μm). DOX+NaB, DOX+NaCl group *n* = 8 per group. Error bars represent the mean ± SEM. Statistical analyses were performed using Student’s *t* tests for two groups and one/two-way ANOVA for multiple comparisons. ∗*p* < 0.05; ∗∗*p* < 0.01; ∗∗∗*p* < 0.001; ∗∗∗∗*p* < 0.0001.

We found that decreased butyrate levels were related to increased susceptibility to DIHF. For further validation, H9C2 rat cardiomyocytes were co-cultured with NaB *in vitro*, and the result indicated that compared to the control group, NaB remarkably increased the viability of H9C2 cardiomyocytes (Figure 3A), In contrast, DOX treatment considerably decreased the viability of H9C2 cardiomyocytes in a concentration-dependent manner (Figure 3B). Next, we tested whether NaB treatment could reverse DOX-induced toxicity. H9C2 cardiomyocytes were pretreated with 250 ng/mL DOX for 24 h and then co-cultured with 100 μg/mL, 250 μg/mL, or 500 μg/mL NaB. As expected, our results revealed remarkably increased viability of H9C2 cardiomyocytes in the DOX+NaB group in comparison with the DOX control group, suggesting a pronounced reversal of DOX-induced toxicity in H9C2 cardiomyocytes (Figure 3C). To verify these results, we repeated this rescue experiment using the AC16 human cardiomyocyte line. As shown in Figure 3D, our results indicated that NaB notably increased the viability of DOX-treated AC16 cardiomyocytes, demonstrating that NaB treatment could consistently reverse DOX-induced toxicity in human cardiomyocytes. Next, the ROS-specific fluorescence probe DCFH-DA was used to measure ROS levels in H9C2 cardiomyocytes. The results showed that, compared with the DOX control group, NaB treatment in the DOX+NaB group significantly reduced DOX-induced ROS levels in H9C2 cardiomyocytes (Figure 3E). The cardiomyocyte death ratio was determined by propidium iodide (PI) staining, and the results revealed that NaB treatment also significantly decreased the DOX-induced cardiomyocyte death ratio (Figure 3F).

To demonstrate the correlation between butyrate and DIHF *in vivo*, we first determined the appropriate concentration of NaB in the DIHF model. The experiment was divided into three groups, including DOX+NaCl group (supplemented with 0.9%(w/w)NaCl in drinking water), DOX+1%(w/w) NaB group and DOX+5%(w/w) NaB group^25^(supplemented with 1% or 5% NaB in drinking water respectively) (Figure S3A). This result revealed clear differences between the two NaB concentrations: Compared with the other two groups, the 5% NaB treatment induced a significant reduction in rat body weight and a marked shortening of survival time, indicating poor tolerance to the 5% concentration. In sharp contrast, the 1% NaB treatment caused only a non-significant alteration in body weight, while significantly prolonging survival time, compared with the DOX+NaCl control group (Figures S3B and S3C). These findings confirmed that 1% NaB is effective and well-tolerated. Thereafter, rats were supplemented with drinking water containing 1% (w/w) NaB in the DOX+NaB group and 0.9% (w/w) NaCl in the DOX+NaCl control group (Figure 3G). Echocardiography was conducted pre- and post-DOX treatment to evaluate the changes in LVEF and LVFS, and our results confirmed that NaB significantly mitigated the decreases in LVEF and LVFS, indicating NaB supplementation significantly ameliorated DIHF (Figure 3H). Additionally, Hematoxylin-eosin (HE) staining revealed no markedly morphological alterations in the spleen, kidney, and liver tissues between the two groups (Figures S3D–S3F). Moreover, no significant changes were observed in the parameters of kidney function (urea and creatinine) and liver function (aspartate transaminase [AST] and alanine transaminase [ALT]) (Figures S3G and S3H). These results indicate that 1% NaB supplementation does not increase the potential toxicity of these organs.

To validate the effect of NaB supplementation, the serum butyrate was examined in both groups via a colorimetric method, and the data confirmed that the butyrate concentration in the DOX+NaB group was markedly higher with NaB supplementation (Figure 3I). To explore the relationship between butyrate and DIHF, Pearson’s correlation analysis was conducted, and the results indicated that serum butyrate concentration was negatively correlated with the decrease rate of the LVEF (Figure 3J). The results further validated that NaB ameliorated DIHF. To further explore the mechanism, we investigated whether NaB supplementation, which had ameliorated DIHF, resulted in ameliorating cardiac structure. Masson trichrome staining results indicated that cardiac tissue fibrosis was remarkably alleviated in the DOX+NaB group compared with the DOX+NaCl group (Figure 3K). In summary, our findings consistently confirmed that NaB treatment reduces DOX-induced cardiomyocyte toxicity *in vitro* and ameliorates DIHF *in vivo*.

### Sodium butyrate reduces gut microbiota dysbiosis and lowers serum total cholesterol levels in the doxorubicin-induced heart failure rats

**Figure 4:**
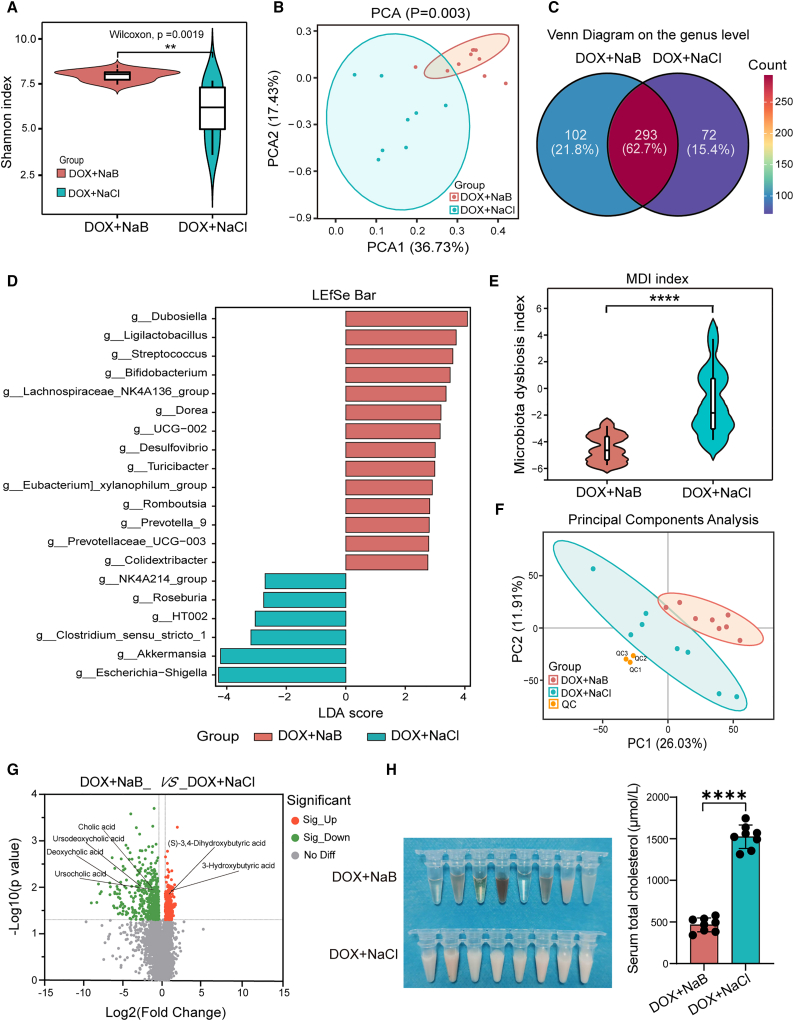
NaB reduced DOX-induced gut microbiota dysbiosis and lowered serum total cholesterol (TC) levels (A) ɑ-diversity (Shannon index) of gut microbiota in the DOX+NaB and DOX+NaCl groups. (B) PCA of gut microbiota in the DOX+NaB and DOX+NaCl groups on the genus level. (C) Venn diagram shows differences in gut microbiota composition on the genus level between the DOX+NaB, DOX+NaCl group. (D) Linear discriminant analysis (LDA) displays the differential gut microbiota in the DOX+NaB and DOX+NaCl groups (LDA score>2). (E) MDI of gut microbiota in the DOX+NaB, DOX+NaCl group. (F) PCA of serum metabolites in the DOX+NaB and DOX+NaCl groups by LC-MS profiling. (G) Volcano plot shows differential serum metabolites in the DOX+NaB, DOX+NaCl group. (H) Serum appearance and serum total cholesterol concentration in the DOX+NaB, DOX+NaCl group. DOX+NaB, DOX+NaCl group *n* = 8 per group. Error bars represent the mean ± SEM. For normally distributed data with homogeneous variances, statistical analyses were performed using Student’s *t* tests for two groups and two-way ANOVA for multiple comparisons. For non-normal/heteroscedastic data, the Wilcoxon rank-sum test was applied for two groups. ∗∗*p* < 0.01; ∗∗∗∗*p* < 0.0001.

NaB supplementation ameliorated DIHF *in vivo*. To elucidate the underlying mechanism, we next explored the effect of NaB on the gut microbiota. We performed a 16S rRNA V3-V4 sequencing on feces from the DOX+NaB and DOX+NaCl groups, and the results revealed that the ɑ-diversity of the DOX+NaB group was considerably elevated compared to the DOX+NaCl group (Figure 4A). PCA indicated that there was a marked discrepancy in gut microbiota composition between the two groups on the genus level (Figure 4B). The Venn plot further showed the discrepancy, with 102 (21.8%) genera specific to the DOX+NaB group and 72 (15.4%) genera specific to the DOX+NaCl group (Figure 4C). LDA revealed that on the genus level, 14 genera were considerably enriched in the DOX+NaB group, 9 of which were probiotic genera. Moreover, 6 genera, 3 of which were probiotic genera, were considerably enriched in the DOX+NaCl group (Figure 4D; Table S1). Importantly, the MDI in the DOX+NaB group was remarkably decreased compared to that in the DOX+NaCl group (Figure 4E). These findings imply that NaB supplementation notably augmented the biodiversity of the gut microbiota and optimized the microbiota composition.

Changes in the gut microbiota composition typically lead to alterations in its biological functions. To demonstrate this, PICRUSt2 analysis was performed. The result indicated that the functions associated with DNA repair and recombination, DNA replication, and primary/secondary acid biosynthesis were considerably enhanced in the DOX+NaB group (Figure S4A). These findings suggested that NaB supplementation regulated gut microbiota composition with the potential to mitigate DOX-induced DNA damage and regulate bile acid metabolism.

The altered gut microbiota composition readily leads to alterations in metabolites. Next, nontargeted metabolomics LC/MS was utilized to analyze the serum metabolites in the DOX+NaB and DOX+NaCl groups. PCA revealed remarkable differences in metabolite composition between the two groups (Figure 4F). As the volcano plot showed, NaB supplementation increased the levels of (S)-3,4-dihydroxybutyric acid and 3-hydroxybutyric acid and decreased the levels of secondary bile acids (Figure 4G). KEGG enrichment analysis revealed that the secondary bile acid biosynthesis (ko00121), fat digestion and absorption (ko04975), bile secretion (ko04976), and gap junction (ko04540) pathways were markedly enriched in the DOX+NaB group in comparison with the DOX+NaCl group (Figure S4B). To determine the impact on lipid metabolism, the serum total cholesterol (TC) levels were measured via ELISA. The data revealed that serum TC levels were remarkably decreased in the DOX+NaB group than in the DOX+NaCl group (Figure 4H). The findings indicated that NaB supplementation modulated lipid metabolism and lowered serum TC levels. In conclusion, NaB supplementation reduced gut microbiota dysbiosis and lowered serum TC levels in the DIHF rats.

### Sodium butyrate reduces bacterial translocation to the heart by enhancing the barrier function of the colonic and cardiac tissues in the doxorubicin-induced heart failure rats

**Figure 5:**
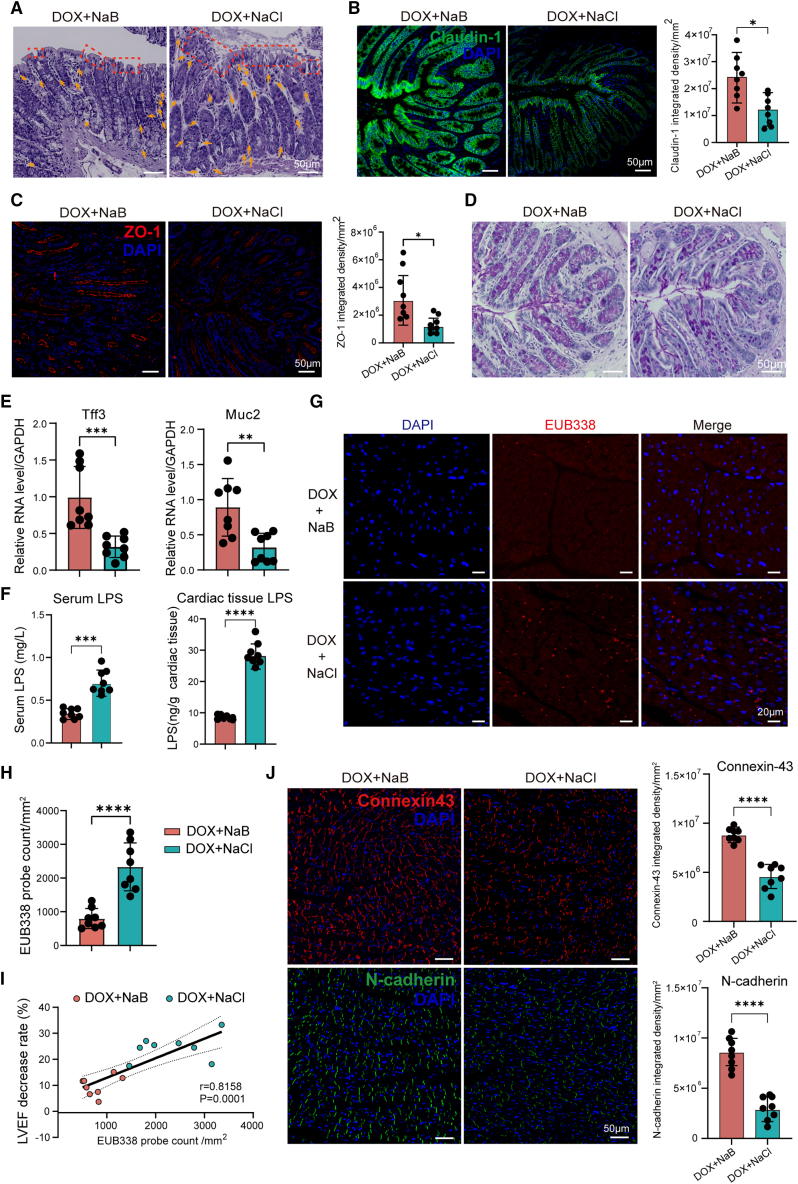
NaB reduced bacterial translocation and LPS levels by enhancing the barrier function of the colonic and cardiac tissues (A) Histopathological damage of colonic tissue in the DOX+NaB, DOX +NaCl group by Hematoxylin and Eosin (H&E) staining (scale bars, 50 μm). (B and C) Representative images, quantitative analysis of Claudin-1 (B) and ZO-1 (C) of colonic tissue in the DOX+NaB, DOX+NaCl group by immunofluorescence (scale bars, 50 μm). (D) Representative images of goblet cell changes of colonic tissue in the DOX+NaB, DOX+NaCl group by AB-PAS staining (scale bars, 50 μm). (E) The mRNA expression levels of Muc2 and Tff3 in the colonic tissue via qRT-PCR assay in the DOX+NaB and DOX+NaCl groups. (F) Serum and cardiac tissue LPS concentrations in the DOX+NaB and DOX+NaCl groups via ELISA. (G and H) Representative images (G), quantitative analysis (H) of EUB338 bacterial probe count in cardiac tissue of the DOX+NaB and DOX+NaCl groups by FISH (scale bars, 20 μm). (I) The Pearson’s correlation analysis of EUB338 probe count and LVEF decrease rate in the DOX+NaB and DOX+NaCl groups. Statistical significance was determined using the simple linear regression of Correlation. (J) Representative images and quantitative analysis of connexin-43 and N-cadherin in cardiac tissue of the DOX+ NaB, DOX + NaCl group by immunofluorescence (scale bars, 50 μm). DOX+NaB and DOX+NaCl groups, *n* = 8 per group. Error bars represent the mean ± SEM. For normally distributed data with homogeneous variances, statistical analyses were performed using Student’s *t* tests for two groups and one-way ANOVA for multiple comparisons. ∗*p* < 0.05; ∗∗*p* < 0.01; ∗∗∗*p* < 0.001; ∗∗∗∗*p* < 0.0001.

We next investigated the mechanism by which oral NaB supplementation modulated DIHF through gut-heart communication. Hematoxylin and eosin (H&E) staining of rat colonic tissues demonstrated a markedly elevated histopathological damage score^26^ in the DOX+NaCl group than in the DOX+NaB group (Figure 5A; Figure S5A; Table S2), this result indicated NaB supplementation substantially alleviated mucosal damage, which consisted with the enhanced gut microbiotic functions of DNA repair, recombination, and DNA replication in the DOX+NaB group (Figure S4A). Moreover, the nontargeted metabolomics (LC-MS) analysis of fecal and serum samples, along with KEGG analysis, consistently revealed that butyrate activated the gap junction pathway (ko04540) (Figure 2E; Figure S4B). For validation, we next examined the expression levels of the tight junction proteins claudin-1 and ZO-1 in colonic tissues via immunofluorescence. The results indicated that NaB supplementation significantly increased the expression levels of claudin-1 and ZO-1 in the DOX+NaB group compared with those in the DOX+NaCl group (Figures 5B and 5C), In addition, we examined colonic goblet cells, which are the first-line physical barrier against microbial invasion and regulate the homeostasis of gut microbiota.^27^ Our results demonstrated that NaB supplementation significantly increased goblet cell numbers (Figure 5D; Figure S5B). Muc2 and Tff3, secreted by goblet cells, are the major macromolecular components of colonic mucus; thus, they are essential for maintaining gut barrier function and homeostasis.^28^^,^^29^ We further investigated the expression levels of Muc2 and Tff3 via qRT-PCR, the results revealed that NaB markedly upregulated Muc2 and Tff3 RNA levels in the DOX+NaB group compared with the DOX+NaCl group (Figure 5E; Table S3). These findings indicated that NaB supplementation remarkably decreased gut leakage.^30^ Therefore, we determined whether the translocation of gut microbiota to cardiac tissue occurred in the context of gut leakage. The level of lipopolysaccharide (LPS), which is a major component of the bacterial cell wall, was measured via ELISA. The results indicated that both the serum and cardiac LPS concentrations were substantially lower due to NaB supplementation (Figure 5F). To directly explore whether there were translocated bacteria in cardiac tissue, we used the universal bacterial probe EUB338 to detect cardiac resident bacteria via FISH, and the result revealed the presence of resident bacteria in DIHF cardiac tissues (Figure 5G). Moreover, quantitative analysis revealed notably lower EUB338 probe count in the DOX+NaB group than in the DOX+NaCl group (Figure 5H). These results implied that NaB supplementation substantially reduced bacterial translocation to cardiac tissue induced by DOX treatment. To demonstrate the correlation between DIHF and resident bacteria in cardiac tissues, Pearson’s correlation analysis was conducted, and the result revealed a notable positive correlation between LVEF decrease and the EUB338 probe count in cardiac tissues (Figure 5I). This result suggested that NaB ameliorated DIHF by reducing cardiac resident bacteria. For further demonstrate the result, we detected the expression levels of the cardiac barrier proteins connexin-43 and N-cadherin, which were remarkably elevated in the DOX+NaB group than in the DOX+NaCl group (Figure 5J). This finding indicated that NaB remarkably enhanced cardiac barrier function to prevent bacterial invasion. Taken together, these findings suggest that NaB reduces bacterial translocation and LPS levels in cardiac tissues by enhancing the barrier functions of both colonic and cardiac tissues.

### Sodium butyrate significantly alters the cardiac bacterial composition and function, leading to decreased Fe^2+^ levels in favor of suppressing ferroptosis in the doxorubicin-induced heart failure rats

**Figure 6:**
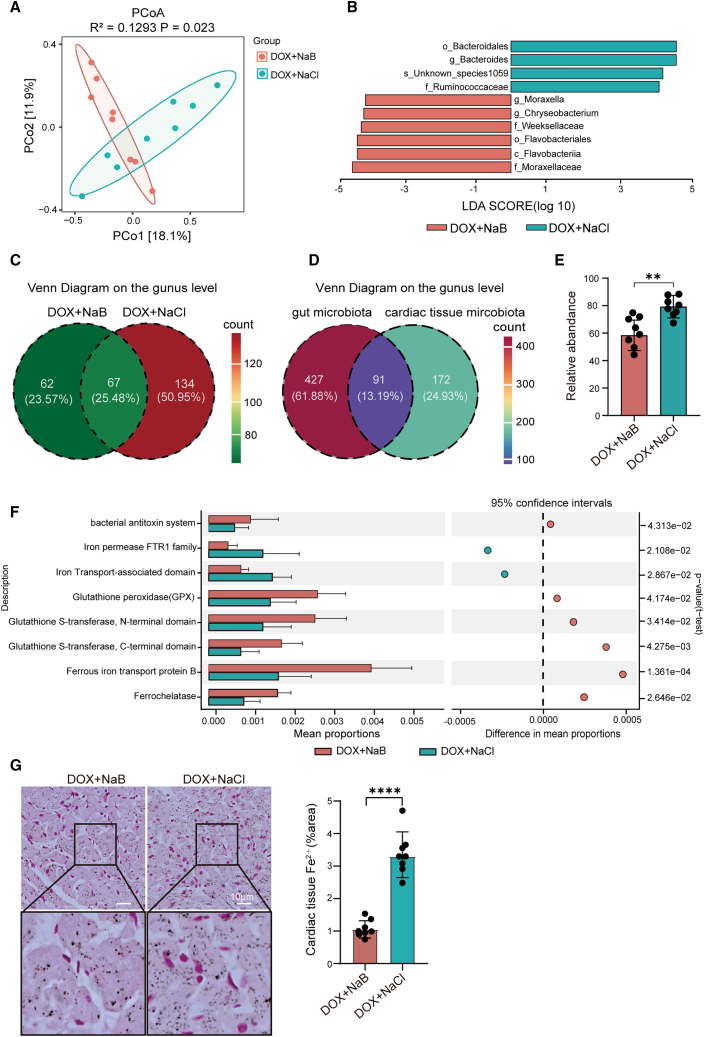
NaB significantly alters the cardiac bacteria composition, leading to bacterial function enhancement and Fe^2+^ level decrease in favor of inhibiting ferroptosis (A) Principal co-ordinates analysis (PCoA) of bacterial composition in cardiac tissue of the DOX+NaB and DOX+NaCl groups by 5R 16s rRNA sequencing. (B) LDA shows differential bacteria in cardiac tissue of the DOX+NaB and DOX+NaCl groups (LDA >3, *p* < 0.05). (C) Venn diagram shows differential bacterial composition in the cardiac tissue of the DOX+NaB and DOX+NaCl groups on the genus level. (D) Venn diagram shows shared bacteria between gut microbiota and cardiac tissue microbiota on the genus level. (E) The relative abundance of translocated gut microbiota within cardiac tissue in the DOX+NaB and DOX+NaCl groups. (F) PICRUSt2 PFAM analysis for the prediction of bacterial functions in cardiac tissue in the DOX+NaB and DOX+NaCl groups. (G) Representative images, quantitative analysis of Fe^2+^ levels in cardiac tissue in the DOX+NaB, DOX+NaCl group. DOX+NaB and DOX+NaCl groups (*n* = 8 per group) (scale bars, 10 μm). Error bars represent the mean ± SEM. For normally distributed data with homogeneous variances, statistical analyses were performed using Student’s *t* tests for two groups and two-way ANOVA for multiple comparisons. For non-normal/heteroscedastic data, the Wilcoxon rank-sum test was applied for two groups. ∗∗*p* < 0.01; ∗∗∗∗*p* < 0.0001.

We determined that NaB significantly reduced DOX-induced bacterial translocation in cardiac tissues. To further elucidate its characteristics, 5R 16S rRNA sequencing (targeting V2/V3/V5/V6/V8 regions) was performed to characterize bacterial communities in cardiac tissues from the DOX+NaB and DOX+NaCl groups. The result revealed remarkable differences in bacterial composition between the two groups by Principal coordinate analysis (PCoA) (Figure 6A). LDA revealed that the abundance of *bacteroides* was markedly elevated in the cardiac tissue of the DOX+NaCl group on the genus level, whereas the abundance of *Moraxella* and *Chryseobacterium* was markedly elevated in the DOX+NaB group (Figure 6B). The Venn diagram showed the distinct bacterial compositions in cardiac tissue on the genus level, and 62 specific genera are found in the DOX+NaB group, and 134 are found in the DOX+NaCl group (Figure 6C). Further analysis revealed that 13.19% (91/690) of the gut microbiota on the genus level were also present in cardiac tissues, accounting for 34.60% of the genera in the cardiac tissue (91/263) (Figure 6D). Moreover, the abundance of translocated gut microbiota within cardiac tissue was remarkably decreased due to NaB supplementation (Figure 6E). Next, PICRUSt2 PFAM analysis was used to predict whether the change of cardiac bacterial composition caused the alteration of the bacterial functions in both groups. The analysis revealed the remarkable enhancement in bacterial functions associated with bacterial antitoxin systems, GPX/glutathione (GSH), and iron metabolism in the DOX+NaB group in comparison with the DOX+NaCl group (Figure 6H). Intriguingly, these bacterial functions were related to ferroptosis, which is an iron-dependent programmed cell death characterized by Fe^2+^-mediated Fenton reaction.^31^ Therefore, we determined the levels of Fe^2+^ in cardiac tissues for preliminary verification; this result indicated that the Fe^2+^ levels were considerably decreased in the DOX+NaB group than in the DOX+NaCl group (Figure 6G). These findings indicated NaB supplementation significantly altered the cardiac bacterial composition, and this alteration caused a remarkable enhancement of the bacterial function and a decrease in Fe^2+^ level in favor of inhibiting ferroptosis in cardiac tissue.

### Sodium butyrate inhibits cardiomyocyte ferroptosis via the GPX4/glutathione pathway in the doxorubicin-induced heart failure rats

**Figure 7:**
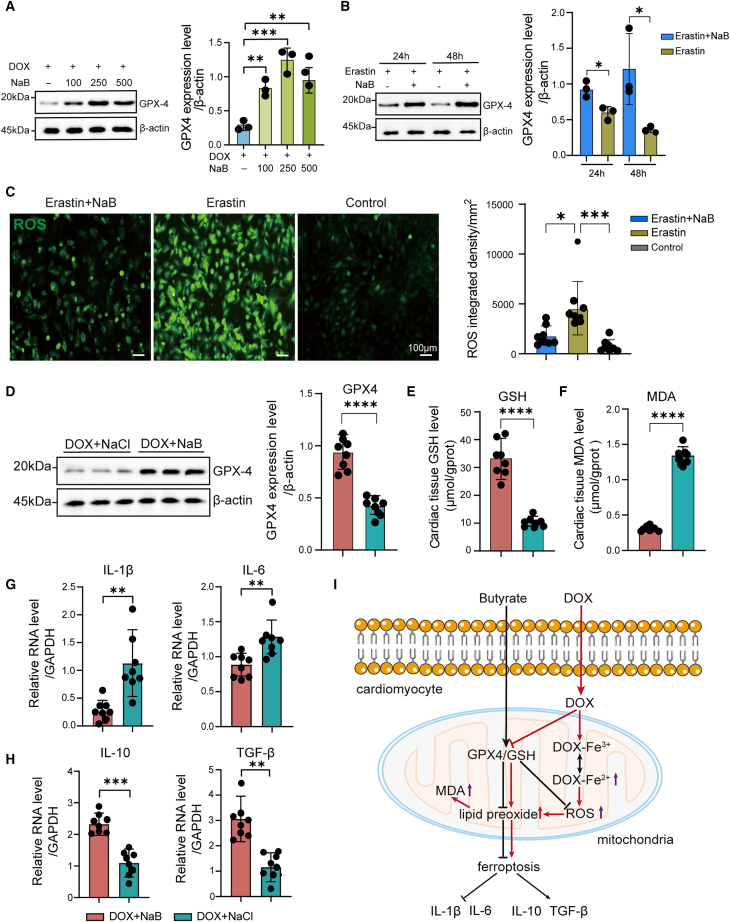
NaB reduced DOX-induced cardiomyocyte ferroptosis via the GPX4/GSH pathway (A) Representative images and quantitative analysis of GPX4 expression levels in H9C2 cardiomyocytes treated with DOX alone, or DOX combined with NaB (100, 250, 500 μg/mL), detected by Western blot assay (*n* = 3 independent experiments). (B) Representative images and quantitative analysis of GPX4 expression levels in H9C2 cardiomyocytes treated with Erastin alone or Erastin combined with NaB for 24 h and 48 h, detected by Western blot assay (*n* = 3 independent experiments). (C) Representative images and quantitative analysis of ROS in Erastin+NaB, Erastin, and control group via DCFH-DA fluorescent probe test (*n* = 8 per group). Statistical significance was determined using the One-way ANOVA test (scale bars, 100 μm). (D) Representative images and quantitative analysis of GPX4 expression levels of cardiac tissue in the DOX+NaB, DOX+NaCl group by Western blot assay. (E) GSH concentration in cardiac tissue of the DOX+NaB and DOX+NaCl groups. (F) MDA concentration in cardiac tissue of the DOX+NaB and DOX+NaCl groups. (G) Change of pro-inflammatory cytokines IL-1β, IL-6 in cardiac tissue of the DOX+NaB and DOX+NaCl groups by RT qPCR assay. (H) Change in anti-inflammatory cytokines IL-10, TGF-β in cardiac tissue of the DOX+NaB and DOX+NaCl groups by RT qPCR assay. (I) The schematic diagram of the mechanism of butyrate inhibiting cardiomyocyte ferroptosis. DOX+NaB and DOX+NaCl group (*n* = 8 per group). Error bars represent the mean ± SEM. Statistical analyses were performed using Student’s *t* tests for two groups and one/two-way ANOVA for multiple comparisons. ∗*p* < 0.05; ∗∗*p* < 0.01; ∗∗∗*p* < 0.001; and ∗∗∗∗*p* < 0.0001.

Our study demonstrated that NaB treatment significantly reduced DOX-induced ROS levels and cell death in H9C2 cardiomyocytes *in vitro* (Figures 3D and 3E). Meanwhile, *in vivo* NaB supplementation considerably decreased cardiac Fe^2+^ level (Figures 6F and 6G). Moreover, DOX has been reported to induce cardiomyocyte ferroptosis, which is characterized by lipid peroxidation driven by ROS accumulation and iron overload, and is primarily suppressed via the GPX4/GSH antioxidant pathway in the mitochondria.^32^ Therefore, we hypothesized that NaB might ameliorate DIHF by suppressing ferroptosis. To validate this hypothesis, H9C2 cardiomyocytes were pretreated with 250 ng/mL DOX for 24 h, followed by treatment with 100, 250, and 500 μg/mL NaB. The expression levels of GPX4, a key inhibitory molecule of ferroptosis, were markedly elevated with NaB treatment in the DOX+NaB group (Figure 7A). Furthermore, we pretreated H9C2 cardiomyocytes with Erastin, a well-established ferroptosis inducer^33^ for 24 h, followed by treatment with 250 μg/mL NaB for 24 or 48 h. The result demonstrated that GPX4 expression level was remarkably elevated due to NaB treatment (Figure 7B). Additionally, NaB treatment significantly reduced Erastin-induced ROS levels *in vitro* (Figure 7C), which promoted lipid peroxidation and thereby induced ferroptosis. Subsequently, we determined the expression levels of GPX4 in cardiac tissue of the DIHF model and found that the expression levels of GPX4 were notably upregulated in the DOX+NaB group compared with the DOX+NaCl group (Figure 7D). Glutathione (GSH), a vital antioxidant of the GPX4/GSH pathway for reducing the lipid peroxidation of cardiac tissue, was markedly increased in the DOX+NaB group (Figure 7E). Conversely, the concentration of malondialdehyde (MDA), a key product of lipid peroxidation, was markedly decreased with NaB supplementation (Figure 7F). These findings confirmed the hypothesis that NaB inhibits cardiomyocyte ferroptosis via the GPX4/GSH antioxidant pathway. Furthermore, Cardiomyocyte ferroptosis triggers inflammatory cascades, promoting the release of pro-inflammatory cytokines^34^^,^^35^ that exacerbate cardiac fibrosis^36^ (Figure 3J) and ultimately lead to heart failure^37^ (Figure 3G). The inflammatory cytokines in cardiac tissue were tested via RT-qPCR assay, the results revealed pro-inflammatory cytokine IL-1β and IL-6 were substantially decreased, while anti-inflammatory cytokine TGF-β and IL-10 were substantially increased in the DOX+NaB group compared with the DOX+NaCl group (Figures 7G and 7H; Table S3). In conclusion, NaB reduced DOX-induced cardiomyocyte ferroptosis through the GPX4/GSH pathway in the DIHF rats (Figure 7I).

## Discussion

Ameliorating DIHF remains a critical challenge in cardio-oncology, necessitating the development of effective and safe adjuvant interventions. This study demonstrated that butyrate, a gut microbiota-derived metabolite, exerts cardioprotective effects against DIHF by inhibiting cardiomyocyte ferroptosis via the gut-heart axis. We first discovered that rats exhibited significant differences in the severity of DIHF during the same DOX dosage treatment. The reason DIHF-HS developed into severe HF was closely associated with decreased butyrate-producing bacteria and butyrate levels. FMT from DIHF-LS to the DIHF model notably mitigated its HF (Figure 1). These findings underscore the critical role of butyrate-producing bacteria and butyrate in modulating DIHF development (Figure 2). It is important that NaB supplementation can modulate the gut microbiota composition and function during DOX treatment, thereby effectively ameliorating DIHF (Figures 3 and 4). Importantly, NaB supplementation strengthened barrier functions in colonic and cardiac tissues, thereby reducing gut bacterial translocation and LPS accumulation in the heart (Figure 5). Interestingly, NaB caused pronounced changes in resident bacterial composition and functions within DOX-treated cardiac tissue, which caused Fe^2+^ levels to decrease in favor of suppressing ferroptosis. We further demonstrated that NaB significantly mitigated DOX-induced cardiomyocyte ferroptosis through the GPX4/GSH antioxidant pathway (Figure 7). Collectively, these mechanisms highlight the crosstalk through the gut-heart axis by which NaB suppresses cardiomyocyte ferroptosis to ameliorate the DIHF.

The correlation between gut microbiota and DIHF has been preliminarily verified.^38^^,^^39^ Our study further demonstrated that DIHF development is related to butyrate-producing bacteria and serum butyrate levels (Figure S1; Figure 2C). NaB supplementation enriched probiotic populations and depleted pathogenic bacteria in the gut; this means that modulating gut microbiota or increasing serum butyrate levels represent a promising cardioprotective strategy against DIHF. Interestingly, NaB supplementation significantly lowered serum TC levels (Figure 4H). Previous studies have shown that gut microbiota dysbiosis elevates cholesterol, which causes cardiovascular diseases.^40^ During HF, cardiac metabolism is converted from fatty acid oxidation to glycolysis and ketone body metabolism as alternative sources of ATP.^41^ Our metabolomics analysis showed that NaB supplementation increases serum levels of 3-Hydroxybutyric acid (Figure 4G), which ameliorates cardiac pathologic remodeling and dysfunction.^42^ Collectively, these results mean that butyrate-producing bacteria and butyrate level were closely associated with improvements in DIHF.

Importantly, NaB also reduced the translocation of gut bacteria and LPS accumulation to the heart by enhancing the barrier function in both colonic and cardiac tissues (Figure 5). These barrier proteins prevent pathogens and harmful substances from infiltrating the bloodstream and into cardiac tissue, thus maintaining cardiac homeostasis.^43^ Ferroptosis is the major form of programmed cell death in DIC.^32^^,^^44^ The microbiota modulates ferroptosis through its microbial composition, metabolites, and biological functions.^45^^,^^46^ Our study is the first to identify resident bacteria in DOX-treated cardiac tissue (Figure 5G). Intriguingly, our study revealed NaB altered resident bacterial composition and biological functions within cardiac tissue, leading to Fe^2+^ level decrease in favor of inhibiting ferroptosis (Figure 6F). We further confirmed NaB inhibited DOX-induced cardiomyocyte ferroptosis via the GPX4/GSH pathway *in vitro* and *in vivo*. NaB inhibited ferroptosis to modulate the release of inflammatory cytokines that alleviated cardiac fibrosis^36^ (Figure 3K) and ultimately ameliorated DIHF^37^ (Figure 3H). Our study further extends the mechanistic understanding that NaB inhibited cardiomyocyte ferroptosis by altering cardiac bacterial composition and biological functions in the DIHF model.

In summary, this study demonstrated that butyrate ameliorated DIHF by inhibiting cardiomyocyte ferroptosis via the microbiota/metabolites-gut-heart axis. Butyrate is one of the short-chain fatty acids (SCFAs) that have been proven to exert protective effects on CVDs and HF.^47^^,^^48^^,^^49^ In this study, we systematically explored the underlying mechanisms of the gut-heart axis by which NaB ameliorated DIHF; this lays the foundation for the clinical translation of butyrate. Surprisingly, more than 80 clinical trials using butyrate as the intervention measure have already been registered on ClinicalTrials.gov. Some trials have already used butyrate to treat patients with cancer or cancer treatment-related complications and achieved favorable clinical outcomes, but it has not been used in CTR-CVT. Based on these studies, it can be sufficiently demonstrated that butyrate has clinical translation potential for treating DIHF in cardio-oncology.

### Limitations of the study

This study has some limitations. First, 16S rRNA gene sequencing usually only allows the classification of gut microbiota to the genus or family level, making it difficult to accurately identify them to the species level. Second, although we used multi-omics analyses to investigate the effects of gut microbiota on DIHF, the specific roles of individual bacterial species require more in-depth investigation. In the future, we will study individual bacterial species that affect the development of DIHF in depth.

## Resource availability

### Lead contact

Further information and requests for resources should be directed to and will be fulfilled by the Lead Contact, Xiaozhong Qiu (qqiuxzh@163.com).

### Materials availability

This study did not generate new unique reagents.

### Data and code availability

• The raw data that support the findings of this study are deposited in the publicly accessible databases. The raw 16S rRNA sequencing reads were deposited in the Sequence Read Archive (SRA) of the National Center for Biotechnology Information (NCBI) (BioProject: PRJNA1189050, PRJNA1189683, PRJNA1189707). The raw LC/MS sequencing reads were deposited in Metabolights (https://www.ebi.ac.uk/metabolights, accession code: MTBLS11830, MTBLS11819). These accession numbers for the datasets are listed in the key resources table. All data reported in this article will be shared by the lead contact upon request.
• This article does not report original code.
• Any additional information required to reanalyze the data reported in this article is available from the lead contact upon request.

## Acknowledgments

This work was supported by the 10.13039/501100001809National Natural Science Foundation of China (Grant Nos. 32430057, U21A20173, 82204417, and 82272977), the 10.13039/501100021171Guangdong Basic and Applied Basic Research Foundation (Grant Nos. 2023B1515120055, 2024A1515011482), and the Guangzhou Science and Technology Program Projects (2025A03J3141).

## Author contributions

S.Z. performed the experiments and the statistical analyses, wrote the article. Q.X. researched the data, performed the statistical analyses, and wrote the article. R.Z. performed part of the experiments and the statistical analyses. D.L performed part of the experiments. T.Y. and J.Z. performed the 16S rRNA analyses. X.Q. and S.M. carried out the conception and coordination of the study, contributed to the discussions, and reviewed the article. Q.X. and X.Q. supplied the funds required for the experiment. All authors approved the final version to be published.

## Declaration of interests

The authors declare no competing interests.

## STAR★Methods

### Key resources table

**Table:** 

REAGENT or RESOURCE	SOURCE	IDENTIFIER
**Antibodies**		

Rabbit GPX4 Polyclonal Antibody	beyotime	Cat# AF7020; RRID: AB_10898356
Rabbit anti-Beta Actin	beyotime	Cat# AF5003; RRID: AB_10700003
Rabbit ZO-1 Polyclonal antibody	proteintech	Cat# 21773-1-AP; RRID: AB_2533456
Rabbit Claudin 1 Polyclonal antibody	proteintech	Cat# 28674-1-AP; RRID: AB_2881190
Rabbit E-cadherin Polyclonal antibody	Servicebio	Cat# GB11868-100; RRID: AB_10697811
Rabbit Connexin 43 Polyclonal antibody	Servicebio	Cat# GB11234-100; RRID: AB_2880711
HRP-conjugated secondary antibody	ZSBG-Bio	Cat# 7077S; RRID: AB_330924

**Biological samples**		

rat feces	This paper	N/A
rat serum	This paper	N/A
rat cardiac tissues	This paper	N/A

**Chemicals, peptides, and recombinant proteins**		

BeyoECL Plus	beyotime	Cat# P0018S
Sodium butyrate	Macklin	CAS# 156-54-7
Propidium Iodide	Solarbio	Cat# C0080
Fetal bovine serum	Vivacell	Cat# C04001
Erastin	beyotime	Cat# SC0224-5mg
DAPI Reagent	Solarbio	Cat# D1306
Doxorubicin	Macklin	Cat# D2975000
Ampicillin	Macklin	Cat# A-301-100
Vancomycin	Macklin	Cat# V-200-25
Neomycin	Macklin	Cat# *N*-620-100
Metronidazole	Macklin	Cat# 443-48-1

**Critical commercial assays**		

Rat Total Cholesterol Colorimetric Assay Kit	Mreda	Cat# M053567
Rat Butyrate Acid Colorimetric Assay Kit	ELK biotechnology	Cat# ELK8174774ELK8174ELK
ROS Assay Kit	beyotime	Cat# S0033S
Rat LPS ELISA Kit	Animaluni	Cat# LV20685
GSH Colorimetric Assay Kit	Elabscience	Cat# E-BC-K030-M
MDA Colorimetric Assay Kit	Solarbio	Cat# E-BC-K025-M
Flash Cell/Tissue Total RNA Kit	YEASEN	Cat# 19221ES50
2×Q3 SYBR qPCR Master Mix	TOLOBIO	Cat# 22204-02
ToloScript All-in-one RT Easy Mix	TOLOBIO	Cat# 22107
BCA Protein Assay Kit	Biosharp	Cat# BL521A
Lillie’s Ferrous Iron Stain Kit	Solarbio	Cat# G3320
CCK-8 Assay Kit	Solarbio	Cat# Ca1210
AB-PAS Stain Kit	Solarbio	Cat# G1285
Amplex Red AST Activity Assay Kit	beyotime	Cat# P2715S
Amplex Red ALT Activity Assay Kit	beyotime	Cat# P2711S
Amplex Red Creatinine Assay Kit	beyotime	Cat# S0291S
Urea Colorimetric Assay Kit	beyotime	Cat# S0574S

**Deposited data**		

The raw 16S rRNA sequencing data	This paper	NCBI Bioproject: PRJNA1189050, PRJNA1189683, PRJNA1189707
The raw LC/MS sequencing data	This paper	Metabolights: MTBLS11830, MTBLS11819

**Experimental models: Cell lines**		

Human AC 16 cardiomyocyte	Millipore	Cat# SCC109
Rat H9C2(2-1) cardiomyocyte	HyCyte	Cat#TCR-C607

**Experimental models: Organisms/strains**		

Rat: Sprague-Dawley	the Experimental Animal Center of Southern Medical University	N/A

**Oligonucleotides**		

EUB338 FISH probe: 5′-GCTGCCTCCCGTAGGAGT-3′	FUBO-Biology	Cat# FBFP130
see Table S3 for RT-PCR Primer	This paper	N/A

**Software and algorithms**		

GraphPad Prism 9 software	Graph-Pad Software, La Jolla, CA, USA	https://www.graphpad.com
Adobe Illustrator 2022 software	Adobe Illustrator software,San Jose, CA, USA	https://www.adobe.com/products/illustrator.html
ImageJ software (1.53t)	National Institutes of Health, USA	https://imagej.nih.gov/ij/
scipy. stats	Python package	https://docs.scipy.org/doc/scipy/
Excel software	Microsoft, USA	https://www.microsoft.com/zh-cn/Microsoft-365/excel
majorbio cloud platform	Majorbio, Shanghai, China	https://cloud.majorbio.com/page/tools/

**Other**		

Immobilon® -P PVDF Membrane	Millipore	Cat# IPVH00010

### Experimental model and study participant details

#### Animal model

All animal experiments in this study were authorized by the Animal Care and Use Committee of Nanfang Hospital, Southern Medical University in Guangzhou, China (SMUL202311025). The experiments were carried out in accordance with the Regulations for Laboratory Animal Management issued by the Ministry of Science and Technology of China. Adult female Sprague-Dawley rats (6–8 weeks old, weighing 200 to 250 g) were procured from the Experimental Animal Center of Southern Medical University and reared in the Animal Experimental Center of the Fifth Affiliated Hospital of Southern Medical University. The rats were kept under fully controlled environmental conditions, including a room temperature of 20–22°C, 50% humidity, and a 12-h light/dark cycle, with unrestricted access to food and water.

#### The susceptibility model of doxorubicin-induced heart failure

The experiment was designed as follows: In the control group, six rats were caged together. In the treatment group, thirty-two rats were caged separately to induce individual differences in gut microbiota for two weeks. After 2 weeks (−14∼0 days), echocardiography (Silicon Wave 60, KOLO, Suzhou, China) was performed before DOX treatment to obtain baseline cardiac function values of LVFS (%) and LVEF (%). Subsequently, the treatment group received DOX (CAS#25316-40-9, Macklin, China) at a dose of 3 mg/kg once a week for a total of five times via intraperitoneal injection (a total dose of 15 mg/kg per rat). The control group was administered 1 mL/kg of 0.9% NaCl solution via intraperitoneal injection. At the 6th week, cardiac function was detected again by echocardiography. Based on the decrease rate of LVEF (1-LVEF_post_/LVEF_pre_), the treatment group was divided into a DIHF-Low susceptibility group (DIHF-LS, with LVEF decrease<20%) and a DOX-High susceptibility group (DIHF-HS, with LVEF decrease>30%). After a 2-week observation period without DOX treatment, cardiac function was again detected at the 8th week by echocardiography. On the 57th day, feces and blood were collected, and the hearts and colons were harvested under anesthesia for further experiments.

#### Fecal microbiota transplantation

To decrease the gut microbiota load in the recipient rats, they were given *ad libitum* access to drinking water containing antibiotic ABX cocktail (0.5 g/L vancomycin, 1 g/L neomycin sulfate, 1 g/L ampicillin penicillin, and 1 g/L metronidazole) for 7 consecutive days(-14∼-7days). All antibiotics were purchased from Macklin Biochemical Technology Co., Ltd. (Shanghai, China). The recipient rats were divided into FMT-DIHF-LS and FMT-DIHF-HS group(*n* = 8),48 h after finishing ABX treatment, the recipient rats were treated with 1 mL donor gut microbiota supernatant by oral gavage, 2 times in initial week(-5days, -3days).next 1 time per week(7∼28days).Before DOX treatment(0 days, pre-DOX treatment), echocardiography was performed to detect baseline cardiac function LVFS (%) and LVEF (%). Then, two groups were administered with DOX 3 mg/kg,1 time weekly with a total of 5 times (total dose 15 mg/kg per rat). After observation for 2 weeks, echocardiography was again performed at the 7th week(42d) to detect cardiac function of post-DOX treatment.

#### NaB supplementation trial in the DIHF rat model

Sixteen adult female Sprague-Dawley rats (6–8 weeks of age, weighing 200–250 g) were housed together for 2 weeks to homogenize the gut microbiota. Following this 2-week (days −14 to 0), echocardiography was conducted prior to doxorubicin (DOX) treatment to acquire baseline cardiac function parameters, including LVFS and LVEF. Thereafter, the rats were randomly assigned to two groups with 8 rats per group: the DOX+NaCl group and the DOX+NaB group. From day 0 to day 28, both groups received intraperitoneal injections of DOX at a dose of 3 mg/kg body weight once weekly, achieving a total cumulative dose of 15 mg/kg per rat. Concomitantly, from day 0 to day 42, rats in the DOX+NaB group were provided with drinking water supplemented with 1% (w/w) NaB (CAS#156-54-7, Macklin, China), whereas those in the DOX+NaCl control group received drinking water containing 0.9% (w/w) NaCl. On day 42, echocardiography was repeated to assess cardiac function and quantify changes in LVEF and LVFS. On day 43, all rats were euthanized, and tissues and blood samples were immediately collected for subsequent analyses.

#### H9C2/AC16 cardiomyocyte culture

The H9C2/AC16 cardiomyocyte were cultured in low glucose(1 g/L) DMEM medium (Cat# C11885500BT, Gibco, USA), supplemented with 10% fetal bovine serum (Cat#C04001, Vivacell, New Zealand)100 U/mL penicillin, and 100 U/mL streptomycin, at 37°C in a humidified atmosphere of 5% CO2 in air.

### Method details

#### Harvesting and processing of feces from donor rats

Feces from DIHF-LS and DIHF-HS were collected, pooled and suspended with pre-chilling phosphate-buffer saline solution (PBS; 100 mg feces/1 mL PBS). Then the samples were homogenized for 10 min to achieve a paste-like consistency, vortexed for 30 s, and centrifuged at 500*g* for 5 min. The supernatant was collected, aliquoted, and stored at −80 °C until transplantation.

#### Fluorescence probe *in situ* hybridization (FISH)

The cardiac tissue sections were dewaxed and rehydrated with a conventional procedure, then were immersed in citric acid (pH = 6.0) and repaired in microwave. After natural cooling, protease K was added dropwise to digest tissue at 40°C, then washing with PBS. Subsequently, the pre-hybridization solution was added dropwise and incubated at 37°C for 1 h. Next, the solution was then dumped, followed by dropping the Probe hybridization solution containing EUB338 probe (1.5:100, FUBO-Biology Cat#FBFP130, China) overnight in the incubator at 40°C. Subsequently, the hybridization solution was washed off at room temperature. Then, DAPI staining solution was added to the slices and incubated for 8 min, the anti-fluorescence quenching sealing agent was added. Finally, the sections were observed and images were collected and analyzed using the ImageJ software (1.53t).

#### Histology and immunohistochemistry

Cardiac and colonic tissue were embedded and sliced by a conventional procedure, H&E and Masson’s trichrome staining was conducted using standard protocols, Fe^2+^ special Staining was performed using Lillie’s Ferrous Iron Stain Kit (G3320, Solarbio, China). Colonic goblet cells were stained with AB-PAS Staining Kit (G1285, Solarbio, China). Images were captured a microscope (DIAN, Hangzhou, China.) and quantified using the ImageJ software (1.53t).

#### Immunofluorescence staining

The paraffin sections were dewaxed, rehydrated, antigen was repaired according to routine protocol. Subsequently, they were incubated with indicated primary antibodies followed by secondary antibodies for immunofluorescence staining, which were goat anti-rabbit IgG of Alexa Fluor 488-conjugated(green) or Alexa Fluor 594-conjugated(red), The slides were then washed three times and stained with DAPI staining solution. The following primary antibodies were used: anti-ZO-1 (21773-1-AP, Proteintech, China), anti-Claudin-1 (28674-1-AP, Proteintech, China), anti-E-cadherin (GB11868-100, Servicebio, China), Anti-Connexin 43 (GB11234-100, Servicebio, China). All the slices were captured with a Nikon ECLIPSE C1 microscope and were processed and quantified by ImageJ software (1.53t).

#### Measurement of MDA, GSH, LPS, butyrate acid, AST, ALT, creatinine, and urea

Commercial ELISA kits were used to measure the concentration of MDA (Cat#E-BC-K025-M, Solarbio, Beijing, China), GSH (Elabscience, Cat#E-BC-K030-M), LPS (Animaluni, Cat#LV20685), Butyrate acid (ELK biotechnology, Cat#ELK81747), AST (Cat#P2715S, beyotime), ALT (beyotime, Cat#P2711S), Creatinine (Cat#S0291S, beyotime), Urea (Cat#S0574S, beyotime), in serum or tissue, according to the manufacturer’s instructions.

#### RNA extraction and real-time PCR of cardiac tissue

According to the manufacturer’s instructions, total RNA was extracted from cardiac tissue using Cell/Tissue RNA extraction Kit (Cat#19331ES50, Yeasen, Shanghai, China). cDNA synthesis was performed using Tolo Script All-in-one RT Easy Mix (Cat# 22107, TOLOBIO, Shanghai, China). Subsequently, The RT-qPCR (Bioer, Hangzhou, China) was conducted using the PCR reaction mixture including 10 μL 2×Q3 SYBR qPCR Master Mix(Cat#22204-02, TOLOBIO, Shanghai, China), 0.4μLfarward primer, 0.4 μL reverse primer, 4 μL cDNA, 5.2 μL ddH2O to a final volume of 20 μL. PCR amplification conditions were set as follows: initial denaturation at 95 °C for 30s, followed by denaturing at 95°C for 10 s with a total of 40 cycles. Relative RNA expression levels were analyzed using the 2-^ΔΔ^Ct method with GAPDH as the internal control.

#### Western blotting analysis

Cardiac tissue was homogenized in ice-cold RIPA lysis buffer, supplemented with protease and phosphatase inhibitor cocktail. H9C2 cardiomyocyte were lysed in RIPA buffer on ice, collected, and ultimately centrifuged to obtain the supernatant. A BCA Protein Assay Kit (BL521A, biosharp, China) was utilized for the determination of the total protein concentration in supernatant. Equal amounts of protein samples were separated on SDS-PAGE gels and then transferred onto PVDF membrane (IPVH00010, Millipore, USA), The membranes were blocked with 5% non-fat milk in 0.1% TBST for 2 h at room temperature and then incubated with primary antibody anti-GPX4(1:1000, Cat#AF7020, beyotime, China), rabbit polyclonal Antibody anti-β-actin (1:2000, Cat#AF5003, beyotime, China) overnight at 4°C. After washing, the PVDF membranes were incubated with the HRP-conjugated secondary antibody (1:3000). Finally, the protein bands were detected with an ECL reagent and visualized using the FluorChem E System.

#### 16S rRNA gene sequencing, data processing and analysis

Fecal samples were collected, stored at −80°C.Total fecal microbial genomic DNA was extracted. The hypervariable region V3-V4 of the bacterial 16S rRNA gene were amplified using primer pairs 338F (5′-ACTCCTACGGGAGGCAGCAG-3′) and 806R(5′-GGACTACHVGGGTWTCTAAT-3'). The PCR reaction mixture included 2 μL 2.5 mM dNTPs, 4 μL 5×Fast Pfu buffer, 0.4 μL Fast Pfu polymerase, 0.8 μL each primer (5 μM), 10 ng of template DNA, and ddH2O to a final volume of 20 μL. PCR amplification were conducted. Subsequently the PCR product was extracted and purified. Purified amplicons were pooled in equimolar amounts and sequenced.

By bioinformatic analysis on the Majorbio Cloud,^50^ Alpha diversity indices and rarefaction curves including Chao1 richness, Shannon index were calculated. PCoA was performed to determine the similarity among the microbial communities in different groups. Differentially abundant genera were identified with LDA effect size (LEfSe, LDA≥3.0, *p* value < 0.05). PICRUSt analysis was used to predict the changes of functional categories using KEGG orthologs.

#### Non-targeted metabolite profiling

Blood samples were collected and centrifuged at 3000 rpm for 10 min, taking the supernatant as serum. Then serums were stored at −80°C for metabolite profiling. Fecal samples were collected, stored at −80°C.Fecal and serum samples was added to mix with grinding bead. Extraction solution was used for metabolite extraction. A pooled quality control sample (QC) was prepared through mixing equal volumes of all samples, disposed and tested in the same manner as the analytic samples.

The LC-MS analysis was conducted.^51^ After the LC/MS raw data pretreated, the metabolites were identified via searching database, HMDB, Metlin. Then, the R package “ropls” (Version 1.6.2) was used to perform PCA and OPLS-DA. The metabolites with VIP>1, *p* < 0.05 were determined as significantly different metabolites. Differential metabolites were mapped into their biochemical pathways based on KEGG database. Python packages “scipy.stats” was used to perform enrichment analysis to obtain the most relevant biological pathways associated with DOX/butyrate treatments.

#### Measurement of H9C2 cardiomyocyte viability, death rate, ROS level

H9C2 cell was treated with appropriate sodium butyrate (CAS#156-54-7, Macklin, China) and DOX. CCK-8 reagent (Cat#Ca1210, Solarbio, China) was utilized to quantify the H9C2 cell viability at 450 nm H9C2 cells were dyed using a Propidium Iodide (PI) solution (Cat# C0080, Solarbio, China) to detect the death cell, and then were stained with DAPI. ROS detection kit (Cat#S0033S, beyotime, China) was utilized to detect the ROS level in H9C2 cell, according to the manufacturer instructions. Images were captured using a microscope and quantified using the ImageJ software (1.53t).

### Quantification and statistical analysis

#### Statistical analyses

The data were presented as mean ± standard deviation (SD), analyzed and plotted using the Graph Pad Prism Software (Version 9.0, California, USA). Data analysis of microbiome sequencing and metabolite profiling were described above. Two-tailed Student’s *t* test was utilized to determine significances between two groups. For the comparisons of more than two groups, one/two-way analysis of variance (ANOVA) was utilized, followed by the post hoc LSD test for multiple comparisons. The relationship between different variables was analyzed by Pearson’s correlation. *p* value < 0.05 was considered to be statistically significant. Statistical significance was defined as ∗*p* < 0.05, ∗∗*p* < 0.01, ∗∗∗*p* < 0.001, and ∗∗∗∗*p* < 0.0001.
