# Supplementary material for: Butyrate ameliorates doxorubicin-induced heart failure by inhibiting cardiomyocyte ferroptosis through the gut-heart axis
Source: iScience. 2026 Jan 20;29(2):114754. doi: 10.1016/j.isci.2026.114754 (PMC12886551; doi:10.1016/j.isci.2026.114754)
Supplement: Document S1. Figures S1–S5 and Tables S1–S3 [file mmc1.pdf]

iScience, Volume *29*

Supplemental information

Butyrate ameliorates doxorubicin-induced heart failure by inhibiting cardiomyocyte ferroptosis through the gut-heart axis

Shibin Zeng, Qing Xie, Rong Zhang, Ting Yang, Dan Liu, Jiani Zhang, Shudong Ma, and Xiaozhong Qiu

Supplementary

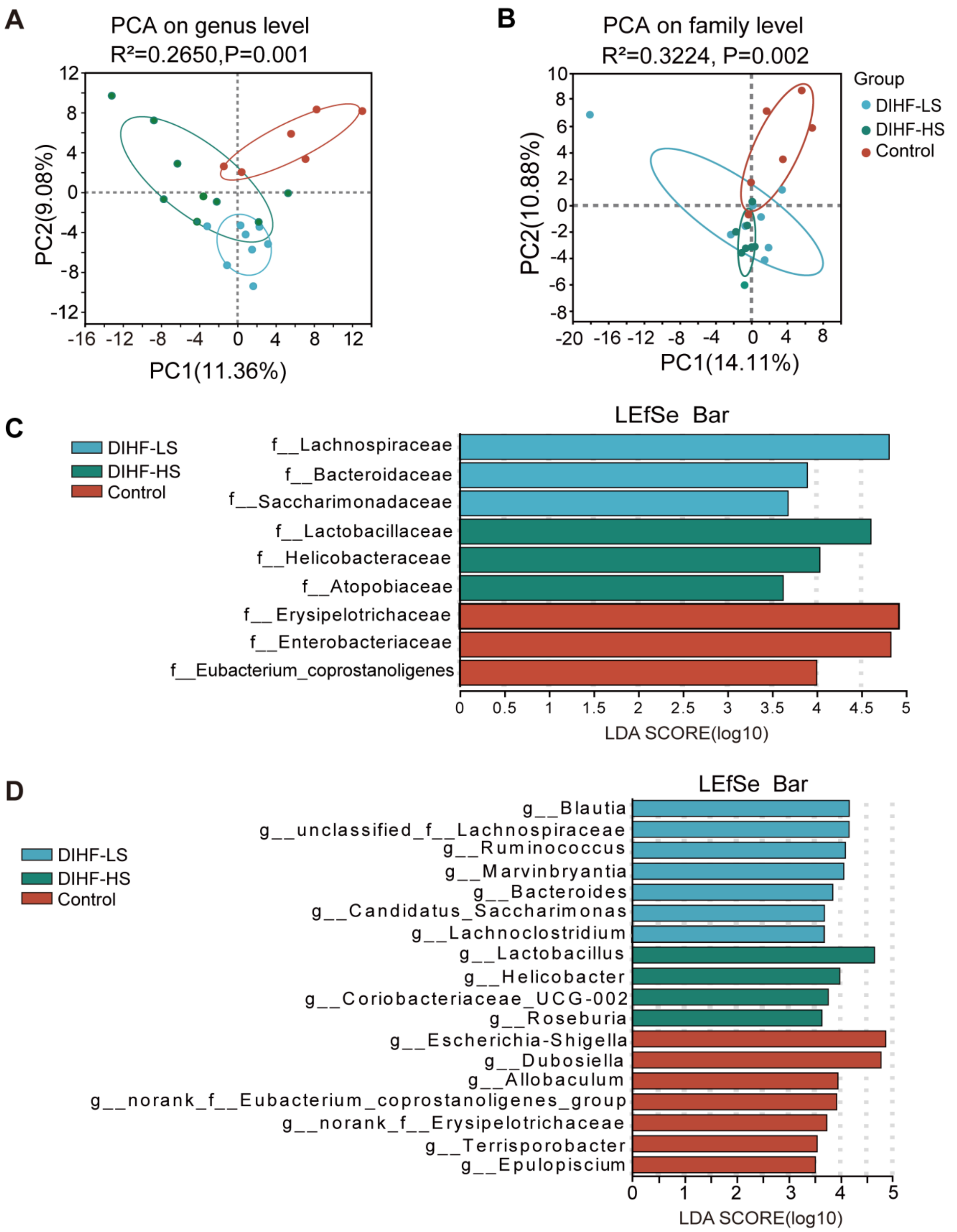

Figure S1. The characteristic of gut microbiota derived from the DIHF-LS, DIHF-HS, Control group.

(A-B) Principal Component Analysis (PCA)of gut microbiota derived from the DIHF-LS,

DIHF-HS, Control group on the genus level(A) and on the family level(B). Data are presented as mean ± SEM.

(C-D) Linear discriminant analysis Effect Size (LEfSe)analysis showing differential abundance of gut microbiota in the DIHF-LS, DIHF-HS, Control group on the family level(C), on the genus level(D).

DIHF-HS group n=9, DIHF-LS group n=8, Control group n=6.

Error bars represent the mean±SEM. For three groups with non-normal distribution or unequal variances, the Kruskal-Wallis test was applied.

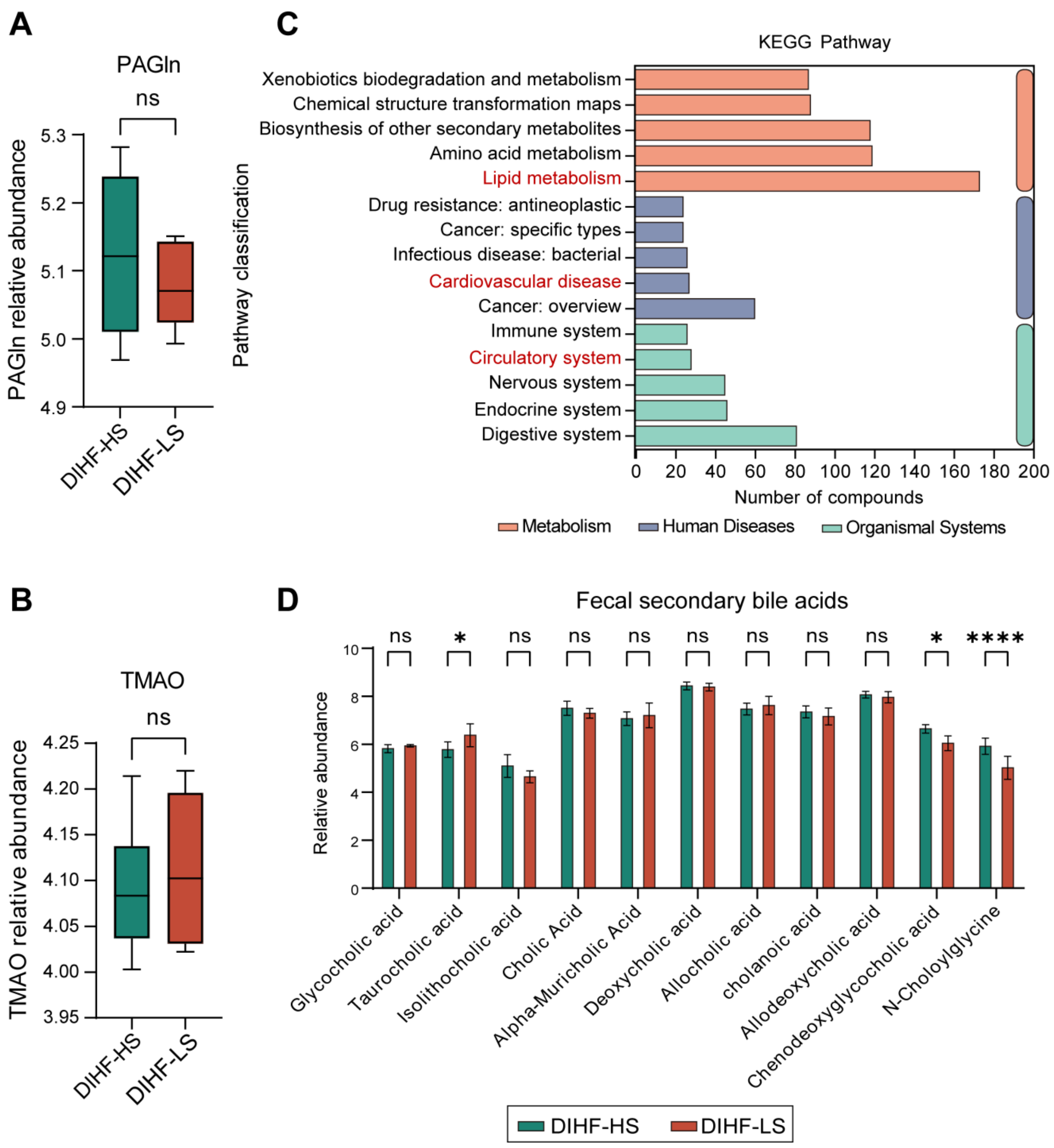

Figure S2. The analysis of fecal LC/MS in the DIHF-HS and DIHF-LS group

(A-B) Relative abundance of fecal trimethylamine N-oxide (TMAO) (A), Phenylacetylglutamine (PAGln) (B) detected by LC/MS in the DIHF-HS and the DIHF-LS group. Data are presented as mean ± SEM. Statistical significance was determined using the Unpaired *t* test, ns, not significant.

(C) Metabolite-related KEGG pathway in the DIHF-HS group compared with the DIHF-LS group.

(D) Relative abundance of fecal secondary bile acids in the DIHF-HS and the DIHF-LS group.

DIHF-HS group n=9, DIHF-LS group n=8.

Error bars represent the mean± SEM. Statistical analyses were performed using Student's t tests for two groups and two-way ANOVA for multiple comparisons. * $p < 0.05$; ****$p < 0.0001$.

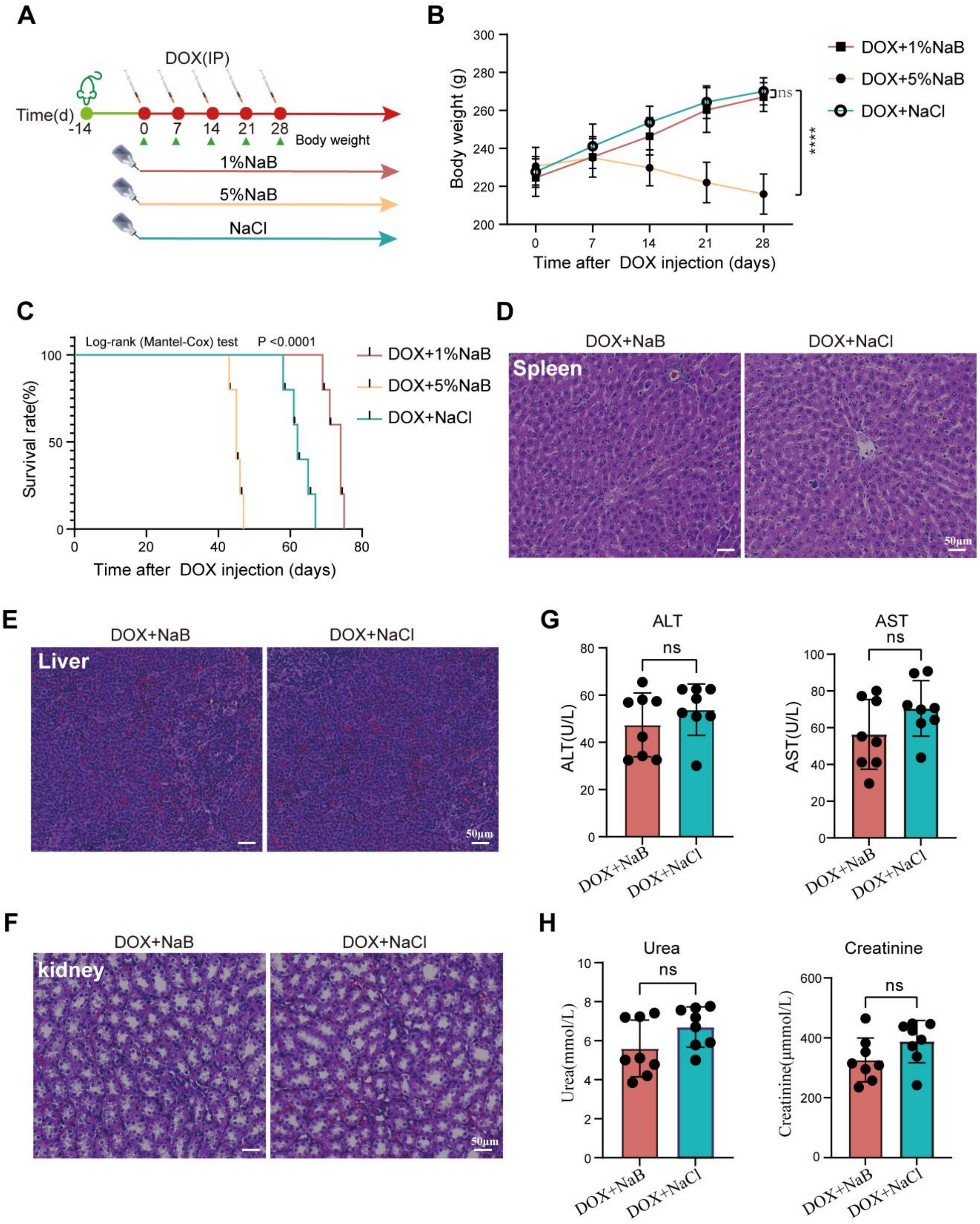

Figure S3. 1% (w/w) NaB exhibited efficacy without increasing potential toxicity to vital organs in the DIHF model

(A) Schematic illustration of the experimental design and timeline for NaB supplementation (at 1% or 5% concentration) in the DIHF model.

(B) The changes of body weight in the DOX+NaCl, DOX+1% NaB, and DOX+5% NaB groups.

(C) Survival time after DOX injection in the DOX+NaCl, DOX+1%NaB, and DOX+5% NaB groups. Statistical analyses were performed using Log-rank (mantel-Cox) test.

(D-F) Morphological change of spleen, liver, and kidney tissue in the DOX+1%(w/w) NaB and DOX+NaCl groups. (scale bar=50μm)

(G-H) Parameters changes of liver function (ALT, AST) and kidney function (urea and creatinine) in the DOX+1% (w/w) NaB and DOX+NaCl groups.

DOX+NaB, DOX+NaCl group n=8 per group

Error bars represent the mean± SEM. Statistical analyses were performed using Student's *t* tests for two groups and two-way ANOVA for multiple comparisons. ****$p < 0.0001$.

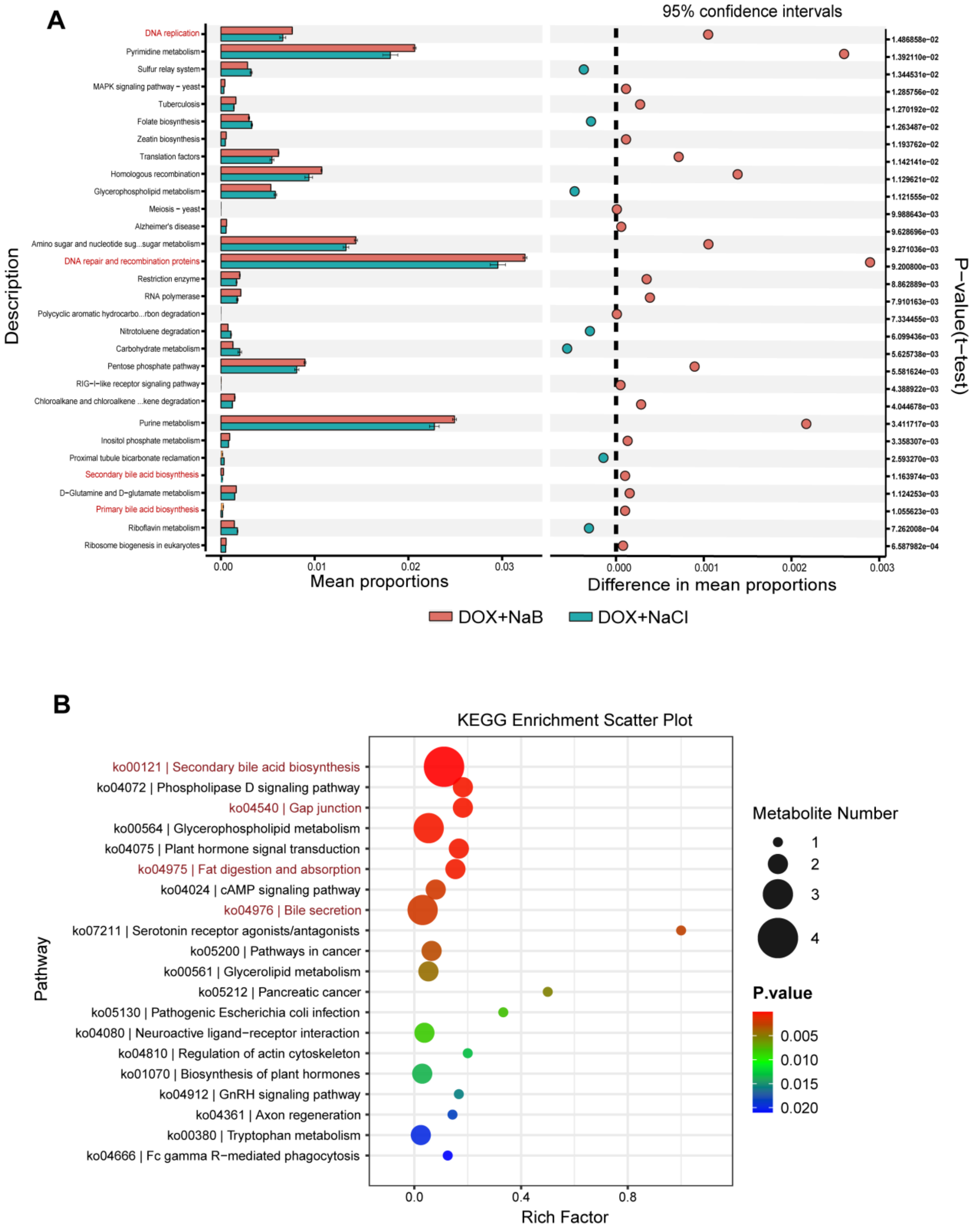

Figure S4. Differential changes of gut microbiota and its metabolite in the DOX+NaB and DOX +NaCl group.

(A) PICRUSt2 analysis showing differential functions of gut microbiota between the DOX+NaB and DOX+NaCl group.

(B) KEGG enrichment analysis showing differential metabolic pathway between the DOX+NaB and DOX+NaCl group.

DOX+NaB, DOX+NaCl group n=8 per group

Statistical analyses were performed using two-way ANOVA for multiple comparisons.

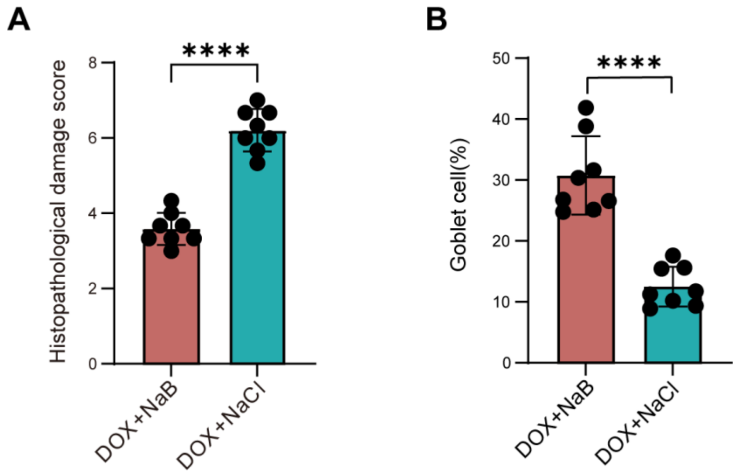

Figure S5. Histopathological analysis of colon tissue in the DOX+NaB, DOX+NaCl group

(A) Histopathological damage score of colonic tissue in the DOX+NaB, DOX+NaCl group by Hematoxylin and Eosin(H&E) staining

(B) Quantitative analysis of goblet cell changs of colonic tissue in the DOX+NaB, DOX+NaCl group by AB-PAS staining

DOX+NaB, DOX+NaCl group n=8 per group

Error bars represent the mean± SEM. Statistical analyses were performed using Student's *t* tests for two groups. ****$p < 0.0001$.

Table S1: The characteristic of differential bacteria of gut microbiota in the DOX+NaB, DOX+NaCl group

group	bacteria	characteristic	taxonomy
DOX+NaB	g_Dubosiella	butyrate/probiotics	Firmicutes/Erysipelotrichaceae
DOX+NaB	g_Ligilactobacillus	probiotics	Firmicutes/Lactobacillaceae
DOX+NaB	g_Streptococcus	pathogen	Firmicutes/Streptococcaceae
DOX+NaB	g_Bifidobacterium	butyrate/probiotics	Actinobacteria/Bifidobacteriaceae
DOX+NaB	g_Lachnospiraceae_ NK4A136_group	butyrate/probiotics	Firmicutes/Lachnospiraceae
DOX+NaB	g_Dorea	butyrate/probiotics	Firmicutes/Lachnospiraceae
DOX+NaB	g_UCG−002	pathogen	Firmicutes/Oscillospiraceae
DOX+NaB	g_Desulfovibrio	opportunistic pathogen	Proteobacteria/Desulfovibrionaceae
DOX+NaB	g_Turicibacter	Probiotics, lowering lipid and bile acid	Firmicutes/Peptostreptococcaceae
DOX+NaB	g_Eubacterium]_ xylanophilum_group	butyrate/probiotics	Firmicutes/Lachnospiraceae
DOX+NaB	g_Romboutsia	butyrate/probiotics lowering lipid and bile acid	Firmicutes/Peptostreptococcaceae
DOX+NaB	g_Prevotella_9	butyrate/probiotics	Bacteroidetes/Prevotellaceae
DOX+NaB	Prevotellaceae UCG-003	opportunistic pathogen	Bacteroidetes/Prevotellaceae
DOX+NaB	g_Colidextribacter	unclear	Firmicutes/unclear
DOX+NaCl	g_NK4A214_group	unclear	Firmicutes/Oscillospiraceae
DOX+NaCl	g_Roseburia	butyrate/probiotics	Firmicutes/Lachnospiraceae
DOX+NaCl	g_HT002	unclear	
DOX+NaCl	g_Clostridium_sensu_ stricto_1	butyrate/probiotics	Firmicutes/Clostridiaceae
DOX+NaCl	g_Escherichia−Shigella	pathogen	Proteobacteria/Enterobacteriaceae
DOX+NaCl	g_Akkermansia	butyrate/probiotics	Verrucomicrobia/Verrucomicrobiaceae

Table S2. The histopathological damage score of colon tissue

Feature	Score	Description
Inflammation level	0	None
	1	Slight
	2	Moderate
	3	Severe
Lesion depth	0	None
	1	Mucosa
	2	Mucosa and submucosa
	3	Transmural
Crypt damage	0	None
	1	Basal 1/3 damaged
	2	Basal 2/3 damaged
	3	Only surface epithelium intact
	4	Entire crypt and epithelium lost
Damage range	0	None
	1	1%-25%
	2	26%-50%
	3	51%-75%
	4	76%-100%

Table S3. The primer sequence for qRT-PCR.

primer	Sequence (5'-3')
IL-1β Forward	ATAGCAGCTTTCGACAGTGAGG
IL-1β Reverse	ACGGGCAAGACATAGGTAGC
IL-6 Forward	CTTCCAGCCAGTTGCCTTCTTG
IL-6 Reverse	TGGTCTGTTGTGGGTGGTATCC
TGF-β Forward	GACCGCAACAACGCAATCTATGAC
TGF-β Reverse	CTGGCACTGCTTCCCGAATGTC
IL-10 Forward	ACTGCTATGTTGCCTGCTCTTACTG
IL-10 Reverse	TGGGTCTGGCTGACTGGGAAG
Muc2 Forward	CTACCACTGCGATGCCAACGA
Muc2 Reverse	GCCACTAACTGCTTGTTCACC
Tff3 Forward	AGGAATTTGTTGGCCTATCTCC
Tff3 Reverse	CGGTTGTTACACTGCTCTGATG
GAPDH Forward	GACATGCCGCCTGGAGAAAC
GAPDH Reverse	AGCCCAGGATGCCCTTTAGT
